# Host identity is the dominant factor in the assembly of nematode and tardigrade gut microbiomes in Antarctic Dry Valley streams

**DOI:** 10.1038/s41598-022-24206-5

**Published:** 2022-11-29

**Authors:** J. Parr McQueen, Kaitlin Gattoni, Eli M. S. Gendron, Steven K. Schmidt, Pacifica Sommers, Dorota L. Porazinska

**Affiliations:** 1 Department of Entomology and Nematology, University of Florida, Gainesville, FL 32611 USA; 2 Department of Ecology and Evolutionary Biology, University of Colorado, Boulder, CO 80309 USA

**Keywords:** Biodiversity, Molecular ecology, Microbial communities

## Abstract

Recent work examining nematode and tardigrade gut microbiomes has identified species-specific relationships between host and gut community composition. However, only a handful of species from either phylum have been examined. How microbiomes differ among species and what factors contribute to their assembly remains unexplored. Cyanobacterial mats within Antarctic Dry Valley streams host a simple and tractable natural ecosystem of identifiable microinvertebrates to address these questions. We sampled 2 types of coexisting mats (i.e., black and orange) across four spatially isolated streams, hand-picked single individuals of two nematode species (i.e., *Eudorylaimus antarcticus* and *Plectus murrayi*) and tardigrades, to examine their gut microbiomes using 16S and 18S rRNA metabarcoding. All gut microbiomes (bacterial and eukaryotic) were significantly less diverse than the mats they were isolated from. In contrast to mats, microinvertebrates’ guts were depleted of Cyanobacteria and differentially enriched in taxa of Bacteroidetes, Proteobacteria, and Fungi. Among factors investigated, gut microbiome composition was most influenced by host identity while environmental factors (e.g., mats and streams) were less important. The importance of host identity in predicting gut microbiome composition suggests functional value to the host, similar to other organisms with strong host selected microbiomes.

## Introduction

Gut microbiomes of many animal species (e.g., aphids, cows, and humans) have been shown to play specific and vital roles in the physiology of their hosts (e.g., digestion of nutrients, protection against pathogens)^1^. In these animals, factors generated by host physical, chemical, or behavioral characteristics deterministically drive the assembly and composition of gut microbiomes in an eco-evolutionary pattern defined as phylosymbiosis^2^. However, hosts’ connection to their microbiomes can vary considerably. For example, gut microbiomes of some animals (e.g., dragonflies and lepidopteran larvae)^3,4^ are more reflective of microbial communities of the hosts’ environment and artificial removal of these microbiomes has no effect on host functioning^5^. Patterns and drivers of gut microbiome assembly can vary substantially, even among closely related host species^5,6^. For example, within the single family of Formicidae, canopy ants (i.e., *Cephalotes*) rely on gut microbiomes to cycle nitrogen and create essential amino acids^7^, but ground ants (i.e., *Solenopsis* and *Pheidole)* are devoid of any gut bacteria^6^ and instead associate with nitrogen rich plants not found in the canopy where the *Cephalotes* reside^8^. This suggests that gut microbiomes of some animals can depend solely on environmental factors and be independent of host identity and/or phylogeny, while other animals might be influenced by a combination of both^9,10^.

Despite our increased understanding of the assembly and function of gut microbiomes of many animals, the microbiomes of nematode and tardigrade species have only recently begun to be studied. Nematoda is an extremely diverse phylum of microscopic roundworms, with estimates of species richness ranging from 0.5 to 10 million, although < 30,000 have been currently described^11^. In addition to taxonomic diversity, nematodes present a wide diversity of life strategies (i.e., r-selected vs. K-selected) and feeding habits (e.g., bacterivores, omnivores, plant parasites, and predators)^12^. Tardigrada is the most closely related phylum to Nematoda^13,14^, and despite significant anatomical differences (e.g., segmented body and 8 legs), there is remarkable similarity in their digestive structures (i.e., a pharyngeal bulb and esophagus), average body size (< 1 mm), molting based growth, feeding habits (e.g., bacterivores, omnivores, and predators), and behavior (e.g., foraging and use of anhydrobiosis), as well as shared feeding niches^15^.

Due to the importance of nematodes in soil processes such as decomposition and nutrient cycling^16^ as well as in agriculture via plant and animal parasitism^17^, nematode gut microbiomes have received more attention than those of tardigrades. Initial studies using DNA clone libraries found that gut microbiomes of several bacterivorous nematode species were characterized by different levels of microbial diversity (e.g.*,* higher in *Acrobeles* sp*.* than *Prionchulus* sp. and higher in *Caenorhabditis elegans* than *Acrobeloides maximus*)^18,19^. With metabarcoding methods, microbial communities of the model nematode *C. elegans* (a bacterivore) have been recently examined and described in more detail^20–24^. It is now well established that the gut microbiome of *C. elegans* is distinct from the microbiome of its natural habitat substrate (rotting fruit)^25^. Moreover, populations of *C. elegans* from widely distant geographic locations with different substrate microbial communities have remarkably similar gut microbiomes^20,22^. In addition, a comparison of the gut microbiome of *C. elegans* to that of its sister species, *C. remanei*, shows compositional differences despite similarity in both feeding habits and environmental niches^20^.

Although gut microbiomes of *Caenorhabditis* species appear to be driven by host identity, the exposure of *C. elegans* to different environmental conditions can affect the composition of the gut microbiome as well, but this remains not fully understood. For example, higher environmental temperatures can result in enrichment of *Sphingobacterium* within the soil substrate but depletion within *C. elegans* guts, while other bacterial taxa (e.g., *Agrobacterium)* can respond in exactly the opposite manner^22^ demonstrating the complex role of environmental factors in shaping the assembly of nematode gut microbiomes. While Tardigrada microbiomes have been studied far less than those of nematodes, they also appear to be host-specific and consistently distinct from their surrounding environment^26–30^. However, varying influence of host identity has been observed within the diverse phylum of Nematoda. For example, while two cryptic *Litoditis* marine species contained distinct microbiomes^31^, a more extensive survey of 33 marine genera found no importance of host identity or feeding habits, suggesting a more stochastic assembly^32^. Unfortunately, nematode and tardigrade gut microbiomes have been characterized for only a handful of species, leaving large gaps in our understanding of the factors driving the assembly of gut communities within the remaining majority of microinvertebrate species that are both phylogenetically diverse and vary greatly in their functional roles within ecosystems.

**Figure 1:**
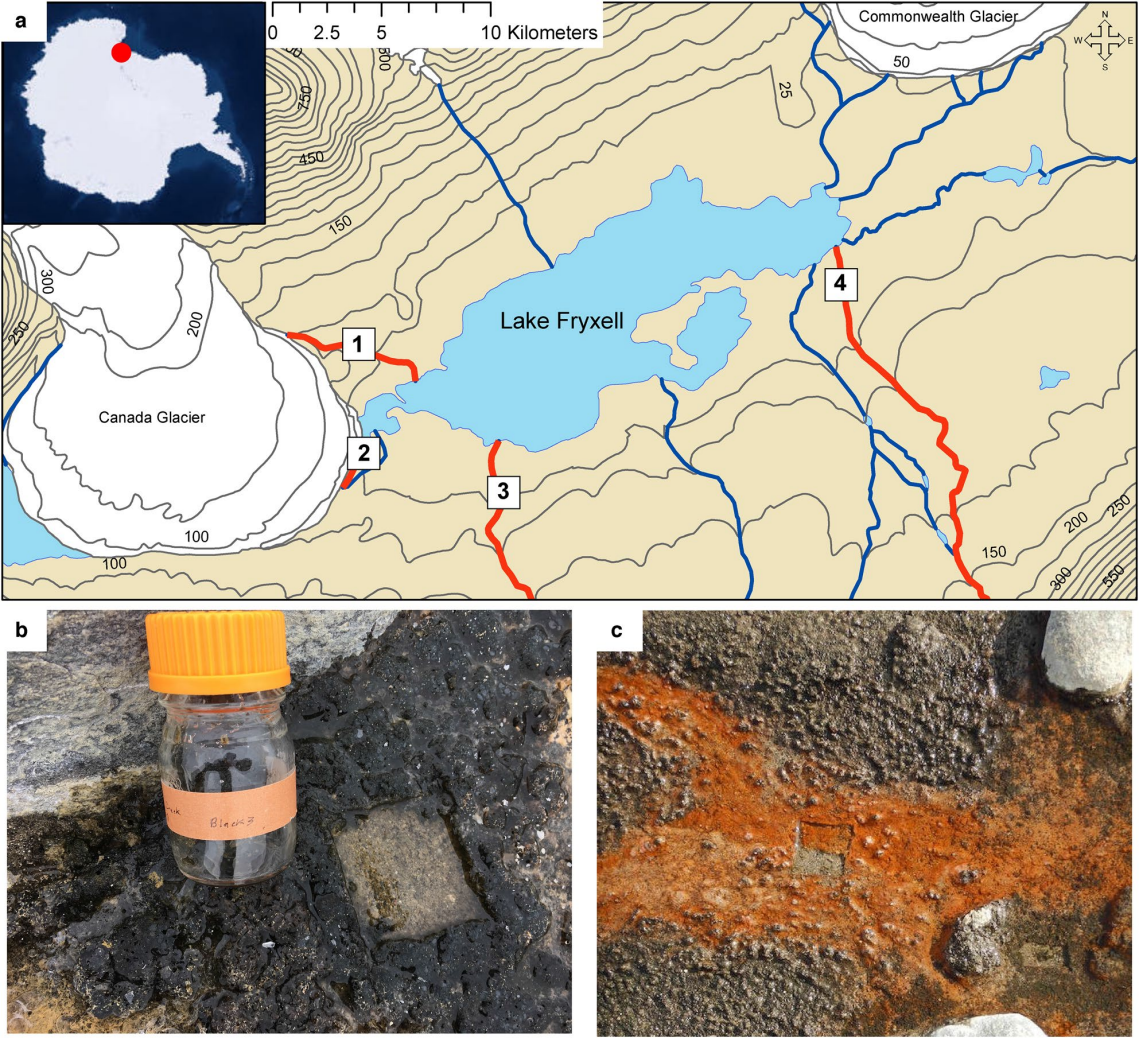
Locations and types of mat samples used for this study. (**a**) Map of the Lake Fryxell Basin in Taylor Valley, Antarctica with exact sampling sites (white squares) along streams (red lines: 1. Canada Stream, 2. Bowles Creek, 3. Delta Stream, 4. Von Guerard Stream). Blue lines indicate other streams not included in this study. Types of mats included in this study: (**b**) black type and (**c**) orange type with orange type showing regrowth 12 months post sampling. Photo credit: Josh Darling and Mike Gooseff. Map was created with ArcGIS Desktop 10.8.2 (http://www.esri.com).

To begin assessing factors driving nematode and tardigrade microbiome assembly more systematically, we used the McMurdo Dry Valleys as a simplified model natural ecosystem. The McMurdo Dry Valleys are an ice-free region of Antarctica, characterized by unvegetated gravel-like soils, extremely high winds, and almost no precipitation. Temperatures within the soil can decline to − 59 °C^33^, restricting continuous biological activity for much of the year. However, during 8–12 weeks of the austral summer, temperatures sufficiently warm to melt adjacent glaciers, generating liquid water that flows down mountain slopes into the basin below^34^. Taylor Valley, the site of this study, contains a number of these seasonally active streams, flowing into a series of permanently ice-covered lakes (e.g., Lake Fryxell) (Fig. 1a). Within the streams reside different types of morphologically distinguishable cyanobacterial mats (e.g., black and orange) (Fig. 1b,c)^35^. Each type of mat is characterized by the dominance of different species of photosynthesizing Cyanobacteria (black by *Nostoc* and orange by Oscillatoriales)^35^ and supports a distinct but diverse microbial community directly adjacent to one another in a single stream^36^. In general, black mats establish at stream margins and orange mats establish within central flows. Both mat types provide a habitat for a finite and well characterized community of microinvertebrates consisting of only two nematode species (*Plectus murrayi* and *Eudorylaimus antarcticus* (currently undergoing taxonomic revaluation)), at least two tardigrade species (*Acutuncus antarcticus* and *Milnesium* sp.), and 3–5 rotifer species^37–39^. Due to its narrow funnel shaped unarmed stoma, *P. murrayi* is classified as a bacterial feeder while *E. antarcticus* contains a hypodermic needle-like odontostylet and is classified as an omnivore^40^. Although *E. antarcticus* has been thought to feed solely on algae^41^, a recent study in Taylor Valley’s Von Guerard stream using isotopic C and N ratios has indicated that while *P. murrayi*, tardigrades (e.g., *A. antarcticus*), and rotifers likely consume only microbes, *E. antarcticus* may rely on food sources consisting of the above mentioned microinvertebrates instead of or in addition to algae^42^. However, the exact food sources and gut microbiomes of these microinvertebrates have not been examined, therefore limiting a full understanding of their roles in the ecosystem.

In this study, we characterized nematode and tardigrade gut microbiomes, as well as the potential factors (e.g., environment such as stream identity and mat type or host-specific factors such as host identity) that could play a role in structuring their assembly. Because this Antarctic ecosystem supports a remarkably simplified and morphologically tractable microinvertebrate community, patterns of gut microbiome assembly can be studied at the nematode species level under natural environmental conditions. In addition to describing the bacterial component of the gut microbiomes, we also examined host-associated microbial eukaryotes. We hypothesized that gut microbiomes of all microinvertebrates would be explained primarily by host identity rather than environmental factors and would therefore be consistent across black and orange mats, and all streams. Consequently, all host associated microbiomes (i.e., bacterial and eukaryotic) would be distinct from the microbial communities of the mats and from each other.

## Results

### Alpha diversity differences among communities

**Figure 2:**
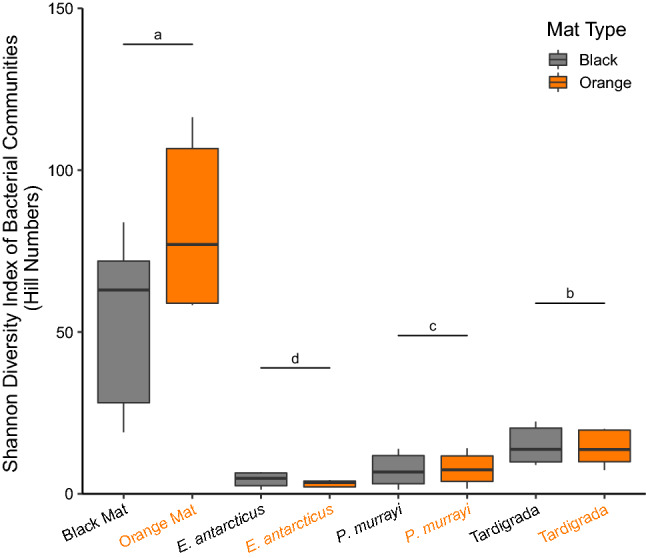
Bacterial ASV Shannon diversity (Hill Numbers) of microbial mats and microinvertebrate gut microbiomes from black and orange mats. Horizontal lines and letters indicate significant difference (*P* < 0.05) among the eight communities with GLM and then Tukey HSD.

Nematode gut microbiomes were assigned into their respective species categories of *E. antarcticus* and *P. murrayi* based on 18S host data that was consistent with morphology (see Methods “Microinvertebrate haplotypes”). In contrast, due to recovery of three undiscernible 18S tardigrade haplotypes, the gut microbiomes were assigned to Tardigrada. Mat bacterial communities were significantly (Tukey's HSD, *P* < 2e−16) more diverse than communities of all microinvertebrate gut microbiomes for all four alpha diversity metrics tested (i.e., Richness, Shannon’s Index, Simpson’s Index, and Faith’s Phylogenic Diversity) (Fig. 2, Supplementary Table S1). In contrast to the significance of community type (i.e., mat, *E. antarcticus*, *P. murrayi*, Tardigrada) for all alpha diversity metrics (GLM, *P* < 0.001, χ^2^(3) > 58.21), there was no effect of mat type (*P* > 0.65, χ^2^(1) < 0.21) on bacterial alpha diversity, while stream was significant for Shannon’s and Simpson’s indices (*P* < 0.01, χ^2^(3) > 11.47) but not for Richness or Faith’s PD (*P* > 0.38, χ^2^(3) < 3.07) (Supplementary Table S1). The interaction between community type and stream was also significant (*P* < 0.001, χ^2^(9) > 23.59) for all metrics tested (Supplementary Table S1a). Gut microbiomes of *E. antarcticus* and *P. murrayi* were less diverse (Supplementary Table S1b) than those of Tardigrada for Shannon’s, Richness, and Faith’s PD (Tukey HSD, *P* < 0.05), but not for Simpson’s (Tukey HSD, *P* > 0.39). Although there was no difference in alpha diversity between nematode species gut microbiomes for Richness, Faith’s PD, and Simpson’s, the Shannon index indicated that gut communities of *E. antarcticus* were the least diverse (*P* < 0.05, Fig. 2).

Within microinvertebrates, most eukaryotic reads predictably assigned to the host (89.25% of *E. antarcticus*, 99.10% for *P. murrayi*, 99.45% of Tardigrada), however when removed, there was sufficient coverage and sequencing depth for further analysis of non-host eukaryotic communities. Mat eukaryotic communities were more diverse than all non-host eukaryotic gut communities for all metrics (Tukey HSD, Richness, Shannon’s, and Faith’s PD, *P* < 1.884e−05; Simpson’s *P* = 0.09) (Supplementary Table S2). Eukaryotic alpha diversity differed between mat types for only Shannon’s (GLM, *P* = 0.06, χ^2^(1) = 3.57) but not for Richness, Simpson’s, and Faith’s PD (*P* > 0.15, χ^2^(1) < 2.10). Among streams, Richness and Faith’s PD were significantly variable (*P* = 0.02, χ^2^(3) > 9.82, Supplementary Table S2a), with Canada Stream being more diverse (Tukey HSD, *P* < 0.05), but not for Shannon’s (*P* = 0.22) or Simpson’s indices (*P* = 0.12) (Supplementary Table S2). Similar to bacterial diversity, the Shannon index indicated that the non-host eukaryotic communities of *E. antarcticus* were the least diverse, followed by *P. murrayi*, and then Tardigrada as the most diverse (Tukey HSD, *P* < 0.05) (Table 2b). For Richness, all gut microbiomes were similarly less diverse than mats, while Faith’s PD showed an overlap of significance for *E. antarcticus* of the other two microinvertebrate communities although *P. murrayi* and Tardigrada did separate from each other (Tukey HSD, *P* < 0.05) (Supplementary Table S2).

### Compositional differences among bacterial communities

Black and orange mats represented two dissimilar microbial communities. Examining only mats, although both mat type (PERMANOVA, *P* < 0.003, *F*(1) = 3.94) and stream (*P* < 0.003, *F*(3) = 4.04) significantly affected the composition of bacterial communities, the communities primarily clustered by mat type (Supplementary Fig. S1a), with only Canada Stream communities separating from those from the other streams (Supplementary Fig. S1b). Consequently, compared to mat type (10%), stream explained the most variation (32%) (Supplementary Table S3a) despite all other streams overlapping in NMDS space (Supplementary Fig. S1b). In contrast, the gut microbiomes of microinvertebrates did not cluster by mat type (Supplementary Fig. S2a) nor by stream, but instead by host identity (e.g., *E. antarcticus*, *P. murrayi*, Tardigrada) (Supplementary Fig. S2b). Although all investigated factors significantly affected gut microbiome compositions (PERMANOVA, *P* < 0.05, *F*(1–6) > 0.48), mat type and stream explained only 1% and 4% of the microbial community variation, respectively (Supplementary Table S4a). In contrast, host identity played the most dominant role in the assembly of gut microbiomes and explained 14% of total variation. However, most of the variation (72%) remained unexplained. At the genus level of taxonomic resolution, 75% of the taxa within Tardigrada, 78% within *P. murrayi,* and 87% within *E. antarcticus* were shared with mats. The remaining proportion of gut taxa was not found within any mats but was in low abundance across all samples. Black and orange mats shared 40% of ASVs, while 27% unique ASVs were assigned to black mats and 33% to orange mats. However, at the genus level, 71% of genera were observed in both mat types with 17% unique genera in black mats and 13% unique in orange mats.

**Figure 3:**
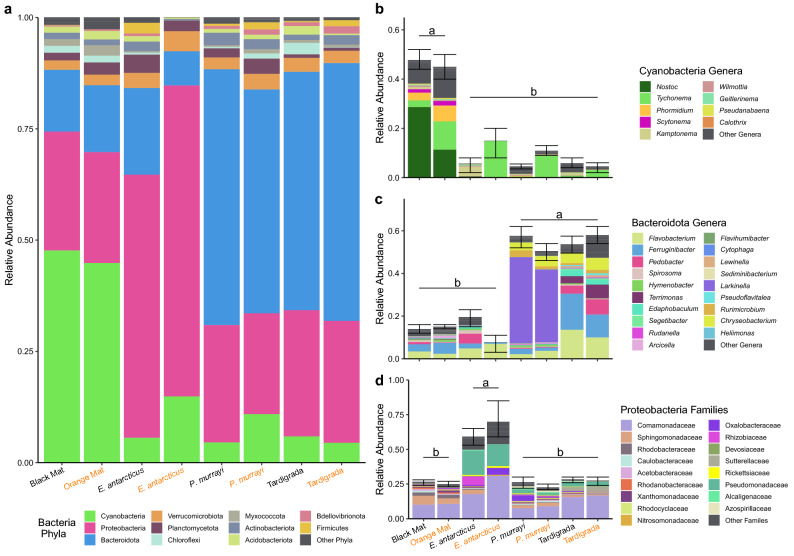
Relative abundance of bacterial communities of mats and microinvertebrate gut microbiomes (**a**) bacterial phyla, (**b**) cyanobacterial genera, (**c**) bacteroidotal genera, and (**d**) proteobacterial families. Horizontal lines and letters indicate statistical differences (*P* < 0.05) at the phylum level among the eight communities tested with a GLMM and then Tukey HSD. Error bars represent SE of entire phylum.

Cyanobacteria, Bacteroidota, and Proteobacteria were the most abundant phyla across all microbiomes comprising 86.40% of the total community composition (Fig. 3a, Supplementary Table S5a). Indicator species analysis confirmed Cyanobacteria, Bacteroidota, and Proteobacteria as significantly indicative phyla of the four microbial community types. Expectedly, Cyanobacteria was the most indicative phylum of the mat communities and although there were six other indicative phyla (Supplementary Table S6), their cumulative relative abundance was low (< 1.2%). Proteobacteria was the sole indicative phylum of the gut microbiomes of *E. antarcticus*. In contrast, Bacteroidota was the sole indicative phylum of the gut microbiomes of *P. murrayi* and was also indicative of Tardigrada. Although Patescibacteria was also indicative of Tardigrada, it comprised < 0.1% of all microbiomes and < 0.28% of Tardigrada gut microbiomes. A phylum of predatory bacterium, Bdellovibrionota, was enriched in all gut types (1.02%) vs. mats (0.2%). Due to both their high proportion within communities, and their significance as indicator species, taxa of the three most abundant phyla were selected for further analysis.

There was no effect of mat type in respect to the relative abundance of the three dominant bacterial phyla, but there was a very strong effect of community type (GLMM, *P* < 1.39e−15, χ^2^(3) > 72.28, Supplementary Table S7a). Streams significantly affected the abundance of Cyanobacteria and Bacteroidota (*P* < 0.001, χ^2^(3) > 14.98), but not of Proteobacteria (*P* = 0.11, χ^2^(3) = 5.93). For Cyanobacteria, both mat types were dominated by a similar overall relative abundance (48% and 45% for black and orange, respectively, Fig. 3a, Supplementary Table S5a), but orange mats contained 70.8% more *Phormidium,* while black mats contained 87.2% more *Nostoc* (Fig. 3b, Supplementary Table S5b). In comparison to mats, all microinvertebrate gut microbiomes were significantly depleted of Cyanobacteria (Fig. 3b) and when compared to each other contained similar amounts of Cyanobacteria. The contribution of cyanobacterial *Nostoc* declined from an average 19.2% within both mat types to 0.2% within gut microbiomes. Although cyanobacterial *Tychonema* was an order of magnitude more abundant than *Nostoc* within gut microbiomes, its relative abundance was more than twice as abundant in all mats (7.5%) than in the guts (3.4%).

Compared to mats, gut microbiomes of *P. murrayi* and Tardigrada*,* but not of *E. antarcticus*, were similarly enriched in Bacteroidota (Tukey's HSD, *P* < 0.05) (Fig. 3c, Supplementary Table S5a). Although gut microbiomes of both *P. murrayi* and Tardigrada were characterized by similar relative abundance of Bacteroidota at the phylum level, at the genus level Tardigrada microbiome taxa were similar to the mats but at larger relative abundances. In contrast, gut microbiomes of *P. murrayi* were distinct from the mats and Tardigrada and significantly enriched by the single genus *Larkinella* making up 37% of its gut community compared to 0.03% of all other microbiome types (Fig. 3c, Supplementary Table S5b). In contrast to Tardigrada, *P. murrayi*, and mats that all contained similar proteobacterial communities, gut microbiomes of *E. antarcticu*s were significantly enriched (Tukey HSD, *P* < 0.05) by Proteobacteria and that of the family Pseudomonadaceae in particular (Fig. 3d). Other Proteobacteria of interest within *E. antarcticu*s included taxa in Rickettsiaceae, a family recognized for its intracellular symbionts. *Wolbachia*, a well-known intracellular bacterium was absent from any microbiome. Comamonadaceae, the most abundant family of Proteobacteria across all communities was almost entirely (97.91%) represented by *Polaromonas.*

**Figure 4:**
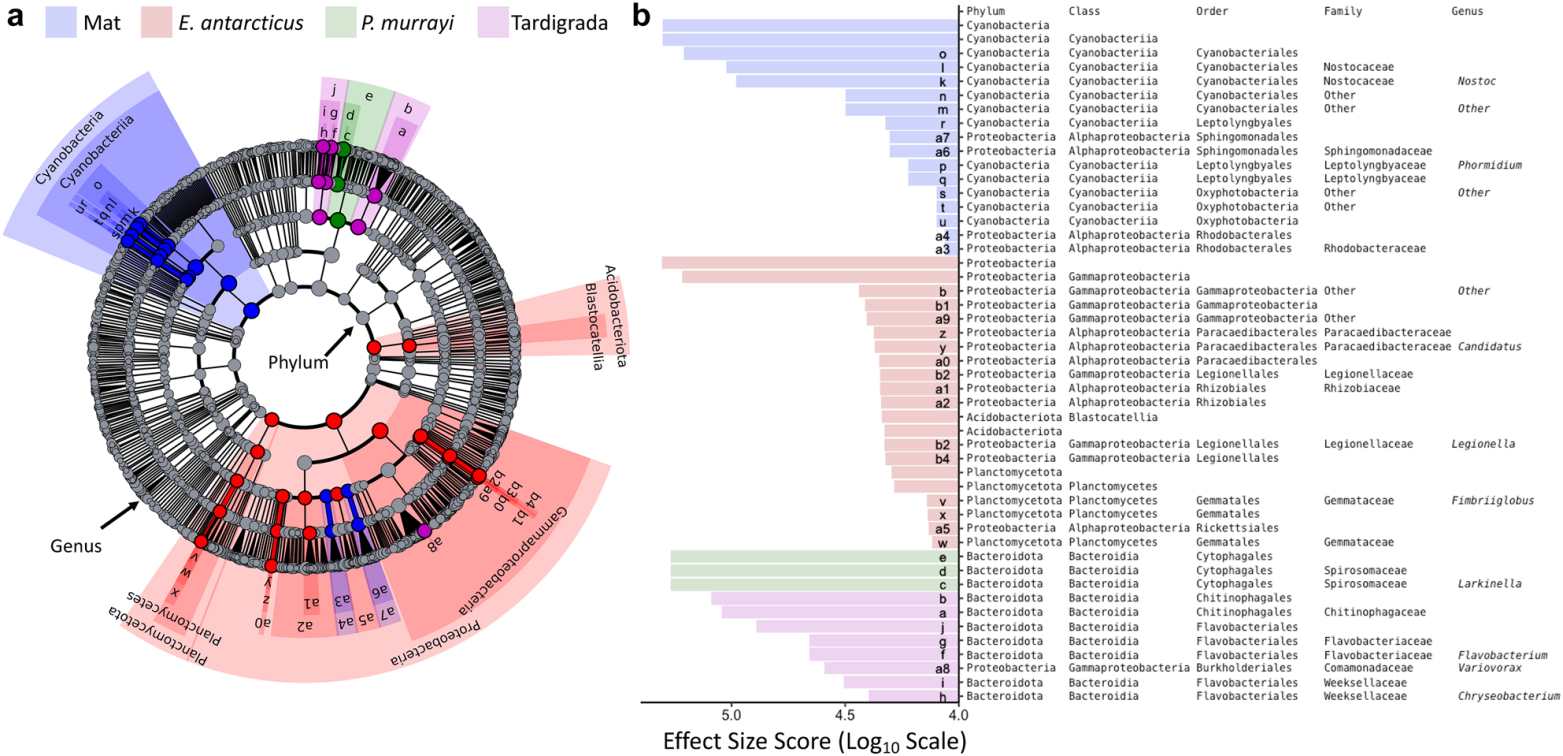
LEfSe (Linear Discriminant Analysis Effect Size) analysis displaying enrichment of most significant bacterial taxa at different levels of taxonomic resolution within mat, nematode (*E. antarcticus* and *P. murrayi*) and Tardigrada microbiomes. Results are visualized in a cladogram with (**a**) circles representing different ranks of taxonomic classification (phylum as the most inner and genus as most outer circle) and community specific significant clades (*P* < 0.05) colored in blue (mats), red (E. *antarcticus*), green (*P. murrayi*), purple (Tardigrada) determined with Kruskal–Wallis and Wilcoxon test. (**b**) LDA effect size scores of each significant clade showing its relative importance in comparison to other significant taxa. Clade identities are written on the cladogram itself or abbreviated and located adjacent to full strings at the base of the effect size scores.

The linear discriminant analysis effect size algorithm (LEfSe) confirmed and further refined these compositional results. LEfSe identified a total of 49 distinctive taxa at different taxonomic ranks specifically affected by the four community types (i.e., mats, *E. antarcticus*, *P. murrayi*, Tardigrada) (LDA effect size = 4, *P* < 0.05) (Fig. 4), but no distinctive taxa when testing for stream (Canada, Bowles Creek, Delta, Von Guerard) or mat type (black or orange). Cyanobacteria (phylum) and Cyanobacteriia (class) were the two most relevant taxa for the mat communities, while Proteobacteria (phylum) and Gammaproteobacteria (subphylum) were the most relevant for gut communities of *E. antarcticus* (Fig. 4b). Acidobacteriota and Planctomycetota were two other indicative phyla of *E. antarcticus* but were lower in effect size score. In contrast, there were no characteristic phylum level taxa of *P. murrayi* nor Tardigrada gut microbiomes. Instead, the most significant taxa of *P. murrayi* gut microbiomes were Cytophagales (order), Spirosomaceae (family), and *Larkinella* (genus) (Fig. 4b), all highlighting taxonomic congruence as Cytophagales and Spirosomaceae are the order and family for *Larkinella*. The five most significant taxa within Tardigrada hosts were all Bacteroidia (class), and not a single taxon was significant above the order level (Fig. 4b). Highlighting the trophic level similarities, indicative taxa for *P. murrayi* and all but one taxon of Tardigrada were within the same class (i.e., Bacteroidia). However, all indicative taxa at the order, family, and genus level were host specific. Of indicative bacterial taxa for all groups, very little overlap of host types was observed among the entire bacterial tree (Fig. 4a), with only five of the 49 taxa of one host microbiome being nested within another.

Clearly distinct microbiomes within mats and microinvertebrates were also supported by preliminary predictions of their functional characteristics. Using PICRUSt2, functional profiles of mat communities (Supplementary Fig. S3) varied primarily by mat type (PERMANOVA, *P* = 0.04, *F*(1) = 2.76), and stream (*P* = 0.07, *F*(3) = 1.79) but stream explained more than twice the variation in the model (21.2% vs 10.9%). Similar to bacterial composition data, microinvertebrate gut microbiome functional profiles (Supplementary Fig. S4) statistically varied by both host identity (PERMANOVA, *P* < 0.01, *F*(2) = 21.10), mat type (*P* = 0.04, *F*(1) = 2.16), and stream (*P* < 0.01, *F*(3) = 8.60). However, host identity was the most important factor in the functional profile as it explained more variation (12.9%) than mat type (< 0.1%) or stream (7.2%) did.

### Compositional differences among eukaryotic communities

Eukaryotic mat communities were dominated by tardigrades, rotifers, nematodes, and algae (Supplementary Fig. S5). Examining the eukaryotic composition of mats, there was a significant difference between mat types (PERMANOVA, *P* = 0.03, *F*(1) = 2.37) and among streams (*P* < 0.001, *F*(3) = 6.18), although stream explained a much larger amount of variation (40.7%) than mat type (5.2%) (Supplementary Table S3b). Canada Stream was particularly important for mat communities, making up half of the overall stream explained variation highlighting the uniqueness of mats from this location. Compositional differences of microinvertebrate non-host eukaryotic communities varied by microinvertebrate type. Although the effects of stream, mat type, and host type were all statistically significant to non-host eukaryotic gut communities (*P* < 0.001, *F*(1–3) > 1.78), similar to bacterial communities, host identity explained the most variation (3.7%), followed by stream (2.5%), and mat type (0.8%) (Supplementary Table S4b). Indicator species analysis identified Metazoa and Chloroplastida as associated with mat communities (*P* < 0.001, R^2^ = 0.69 and 0.43 respectively), while all three microinvertebrates shared the same importance of Fungi (*P* < 0.001, R^2^ = 0.69), at the kingdom level of taxonomic ranking.

Among microinvertebrate gut communities, Fungi comprised the largest (67.3%) component of each microbiome, followed by microbial eukaryotes (including the SAR supergroup), and metazoans (Supplementary Fig. S5). In comparison to mats, all microinvertebrate gut microbiomes were significantly (Tukey HSD, *P* < 3.54e−07) enriched in Fungi (3.3% vs. 48.9–78.5% respectively) (Supplementary Fig. S5b), resulting in a corresponding change in overall community composition. The relative abundance of Fungi in *E. antarcticus* was significantly lower (*P* < 0.01) than in *P. murrayi* and Tardigrada (48.9%, 76.7%, 78.5%, respectively) (Supplementary Fig. S5b). In contrast to mat types, streams affected the fungal abundance within guts (GLMM, *P* = 0.03, χ^2^(3) = 9.00, Supplementary Table S7). Compared to mats, all gut microbiomes were dominated by the subphylum Pezizomycotina and depleted of Blastocladiomycota (Tukey HSD, *P* < 0.05) (Supplementary Fig. S5b). Eurotiomycetes and Leotiomycetes were the most abundant fungal classes (15.7% and 9.5%), and all fungal communities of microinvertebrate guts contained a high diversity of taxa that were compositionally similar.

Metazoans significantly differed in relative abundance among community types (GLMM, *P* < 0.01, χ^2^(3) = 84.74), but there was no influence of mat type or stream (*P* > 0.47, χ^2^(1,3) < 2.51, Supplementary Table S7c). Non-host metazoan ASVs were significantly more common in the gut microbiome of *E. antarcticus* than *P. antarcticus* and Tardigrada (Tukey HSD, *P* < 0.001, Supplementary Fig. S5a). *E. antarcticus* eukaryotic communities were mostly of tardigrade (22.9%) and rotifer origin (5.2%) (Supplementary Fig. S5c). Interestingly, rotifer ASVs were detected in all microinvertebrate guts at similar relative abundances (Tukey HSD, *P* > 0.32). *P. murrayi* reads were detected in only a single specimen of *E. antarcticus*, and in seven out of 94 Tardigrada samples *P. murrayi* reads were in extremely low abundance (< 0.1%)*.* No reads of *E. antarcticus* were found in any guts of *P. murrayi* nor those of tardigrades.

### Microinvertebrate haplotypes

As expected, *E. antarcticus* and *P. murrayi* host 18S ASV data indicated a single species of each across all samples. However, host 18S ASV data indicated the potential presence of three molecular haplotypes of tardigrades all with equal assignments to known Dry Valley tardigrades (possibly Hypsibiidae *Acutuncus antarcticus* or Macrobiotidae *Richtersius* and *Paramacrobiotus*, or Milnesiidae *Milnesium*). None of the alpha diversity metrics tested nor the relative abundance of the three dominant bacterial phyla (i.e., Cyanobacteria, Proteobacteria, Bacteroidota) varied among the three haplotypes (GLM, GLMM, *P* > 0.43, χ^2^(2) < 4.21) (Supplementary Fig. S6). Although the overall bacterial community composition did statistically vary with stream and haplotype explaining 9.9% and 8.1% variation, respectively, they ordinated with complete overlap in NMDS space (Supplementary Fig. S7). Therefore, three possible host haplotypes were combined for this study due to undiscerned haplotype identity.

## Discussion

Our study expands the phylogenetic coverage of nematode and tardigrade gut microbiomes as well as provides new insights into the significance of specific factors of their assembly. While many animals develop well-defined microbiomes, this pattern is not universal. Instead, microbial gut communities may be reflective of the surrounding environment, be random, or even absent^5^. Given that the number of described microbiomes of nematodes and tardigrades thus far is limited, which factors might drive their gut assembly have been hard to predict. Due to the importance of nematodes and tardigrades in ecosystem functioning^16^, it is critical to examine a wide range of hosts from different habitats.

As we hypothesized, microinvertebrate gut microbiomes were better explained by host identities than by environmental factors (e.g., mat type, stream). We observed that all gut microbiomes were less diverse and compositionally distinct from the microbial community of the environment they inhabited for both bacterial and eukaryotic components. At a coarse level of taxonomic resolution (phylum), indicator analysis and statistical tests of relative abundance suggested that microbiomes of bacterivorous *P. murrayi* and tardigrades were similar, and both were significantly distinct from the omnivorous *E. antarcticus* potentially indicating a role of feeding traits on gut microbiome assembly. However, at a finer level of taxonomic resolution, the LEfSe algorithm and relative abundance of orders, families, and genera showed that host identity was the most dominant factor in the assembly of nematode and tardigrade gut microbiomes. Although the potential role of environmental factors was also observed (e.g., mat type and stream), these factors explained less variation than the identity of all three microinvertebrates. These results are largely in line with the literature on *C. elegans* indicating the presence of a conserved gut microbiome that is relatively independent from their surrounding substrate^25^. Interestingly, previously described guts of *C. elegans* have been dominated by Proteobacteria^25^ rather than the Bacteroidota that dominated the guts of *P. murrayi* examined here. Although both nematode species are bacterivores, their different habitat preferences (rotting fruit vs. aquatic cyanobacterial mats) and perhaps phylogenetic placement (Rhabditida vs. Plectida) could be more important in the assembly of gut microbiomes than feeding traits. Nevertheless, Proteobacteria was still a major component of the *P. murrayi* microbiome. Proteobacteria was also the dominant gut component of the omnivorous *E. antarcticus,* similar to the sole other study of a terrestrial omnivorous nematode *Dorylaimus stagnalis*^43^, suggesting its possible importance to the entire nematode phylum. Tardigrade gut microbiomes were even more enriched in both Bacteroidota and Proteobacteria than in previous reports^26,27^ suggesting a functional role of these two bacterial phyla in nematode and tardigrade gut microbiomes. *Flavobacterium* and *Ferruginibacter* were the two most abundant genera of Bacteroidota across all microbiomes, as has been observed in other Antarctic tardigrades^26^ and specifically fully fed (i.e., not starved) Antarctic tardigrades^30^, suggesting this as a reoccurring food source. Interestingly, the LEfSe algorithm identified no phylum level significance for *P. murrayi* or tardigrades, but it did for *E. antarcticus*. This supports the idea of *E. antarcticus* as an omnivore not only for feeding on a wide range of food categories (i.e., fungi, bacteria, algae, micrometazoa), but also as a generalist feeding of taxa within each feeding category (i.e., many types of bacteria). However, individual *E. antarcticus* were less diverse than all other microinvertebrates, indicating that although a population of *E. antarcticus* can feed on a wide range of taxa, individuals do not.

The abstraction of “host identity” likely involves a range of species-specific factors that together may influence the assembly of gut microbiomes. For example, although many nematodes and tardigrades are generally categorized as bacterivorous at the family or genus level, species-specific feeding habits^44^ could impose selective pressure on gut microbiome assembly. This could be explained by the fact that any potential component of gut microbiomes must first pass through the animal’s mouth likely acting as a selective barrier against certain bacterial taxa, with the size of an animal’s stoma imposing a layer of selection against bacteria too large to enter the mouth and digestive system. Easier intake of smaller bacteria by *C. elegans*^45–47^ would support this notion. Likewise, the depletion of large sized *Nostoc *(~ 4 μm diameter per bacterium^48^ and 2 cm–20 cm for colonies) from all microinvertebrate guts in our study could be explained by a small stoma size (~ 3 μm diameter for *P. murrayi*^49^), although it is unclear if *E. antarcticus* could pierce the larger cells with its odontostylet. Nematode esophageal morphology may also play a role in what can colonize the gut instead of simply passing through the nematode. For example, TEM imaging of *C. elegans* and *P. pacificus* cultured on *E. coli* showed broken vs. unbroken *E. coli* cells, respectively due to the presence vs. absence of an esophageal grinder^50^. Since *P. murrayi* contains an esophageal grinder, any bacteria able to colonize the gut must be small and/or resistant enough to maintain viability through this maceration step.

In addition to the filtering of communities resulting from morphological constraints, behavioral factors could also be important. For example, using food preference assays, it has been shown that *C. elegans* can discriminate between higher and lower quality (C:N ratios) bacteria^46^. This ability to discriminate among the quality of their bacterial food sources has been even observed for nematodes incapable of feeding on bacteria (e.g., plant parasitic nematodes)^51^, suggesting food quality sensing is an important factor to consider for all nematode microbiomes. However, it is important to remember that nematodes can be cultured on bacteria that do not colonize the gut. Other poorly understood behavioral factors may also play a role. For example, *Plectus* species from the Antarctic coastline showed preferences for feeding on proteobacterial *Polaromonas*^52^, the most abundant taxon of the family Comamonadaceae observed in all our microinvertebrates as well as mats. In addition, *Polaromonas* has been previously reported within Antarctic tardigrade microbiomes^30^, suggesting that *Polaromonas* may be a common food source for Antarctic microinvertebrates. Unfortunately, apart from a handful of feeding assay studies, very little is known about actual feeding preferences of most nematodes and tardigrades, hindering our understanding of their role in ecosystem functioning. Gut microbiome studies offer a novel mechanism to expediently expand this knowledge as has been done for other animals^53^.

Not everything that passes into the nematode or tardigrade gut can colonize the host and instead some bacteria may be nothing more than transient contents. Although it is methodologically hard to distinguish between the two, it has been shown that specimens of *C. elegans* with established diverse microbiomes in the natural environment maintain their gut microbiomes even when transferred to a culture of *E. coli*^20^, suggesting that once established, nematode gut microbiomes can be stable and resistant to change. A small proportion of gut microbiome taxa were intriguingly absent from mats (13% of genera within *E. antarcticus*, 22% of *P. murrayi*, 25% of tardigrades), although they were all in low abundance and of common phyla. However, it is possible that their lack of presence in mats was an artifact of the lower number of analyzed mat samples (24) than guts (251). Overall, all microinvertebrate species in this study displayed species-specific bacterial microbiomes with little variation across streams and mats suggesting that gut communities examined in this study were composed of actual resident microbes, but further study in this area is warranted.

*Wolbachia*, a common intracellular parasite sometimes reported in nematodes and tardigrades^28^ was not found within any microinvertebrate guts in this study. However, other intracellular bacteria within the sister family of Rickettsiaceae were detected, albeit at very low abundance. We also observed that *Larkinella* sp. was both the most abundant and the most indicative of any bacteria within *P. murrayi* guts. Not much is known about the function of this genus, but isolates grow into pink, horseshoe shaped cells^54^, opening up the possibility for future in vitro studies. Currently however, any possible symbiotic function, or reason *Larkinella* may be so enriched in *P. murrayi* guts is unknown.

All three microinvertebrates sampled in this study contained eukaryotic gut microbiomes primarily consisting of fungi. Fungal microbiomes (i.e., mycobiomes) have only been reported once for marine nematodes^32^ and once for tardigrades^30^, both suggesting well developed mycobiomes. Our data shows that the most abundant fungal ASVs of all hosts were represented by ascomycotous taxa such as *Tetracladium furcatum* and Pyronemataceae, both aquatic fungi known to feed on decomposing matter in streams. While the presence of fungi in the microbiome of an omnivorous nematode (*E. antarcticus*) is not surprising, it is interesting that the presumed bacterial-feeding *P. murrayi* contained higher levels of fungi than the omnivore. This could indicate that previously assigned nematode feeding categories may not be reflective of the exact nematode functional roles in the ecosystem, as *P. murrayi* appears to be intaking fungi even if it does not derive nutrition from it. Interestingly, fungal communities between all microinvertebrates guts were mostly similar, but distinct from previously recorded tardigrade mycobiomes from Italian glaciers^30^, suggesting a possible geographic role of community assembly. In our analysis of non-host eukaryotic gut contents, we found evidence to support the presumption of *E. antarcticus* as the sole omnivore/predator in this ecosystem. Its gut microbiome was characterized by the highest relative abundance of metazoan ASVs, mostly originating from tardigrades and rotifers. Although both *P. murrayi* and tardigrades did contain low relative abundance of other metazoans, especially rotifers, this is most likely a result of the filtering of free-floating DNA from broken cells rather than direct predation. Furthermore, there was no evidence of contamination in the negative controls. High numbers of *P. murrayi* reads observed within a single *E. antarcticus* gut could indicate that *E. antarcticus* may not consistently feed on other nematodes but retains the capability to do so.

Although host identity explained the largest proportion of microbiome variation in this study, most of the variation remained unexplained suggesting other factors may be at play including abiotic environmental factors, stochasticity, biogeography, or inter-microbiome interactions. Prior research of *C. elegans* has shown the presence of interbacterial competition within guts^55^, with greater substrate diversity resulting in less stochastic gut microbiomes^56,57^. Nevertheless, we show that the host identity (rather than feeding traits) relative to environmental factors measured is likely among the more important factors shaping the gut microbiomes of nematodes and tardigrades living in the Antarctic Dry Valleys streams. Although limited in coverage, the functional profiles referred from PICRUSt2 were in line with compositionally unique microbiomes suggesting their differential functional roles in this ecosystem. However, varying influence of host identity has been observed for Nematoda and so to confirm the role of host identity and any functional roles, additional studies using a wider range of species from a diverse range of geographic locations are needed.

## Conclusions

Nematode and tardigrade gut microbiomes have only been described in a select few species, underrepresenting the wide phylogenetic and trophic diversity within these phyla. Of these, even fewer studies have been conducted to examine what drives the assembly of these microbial communities. Using cyanobacterial mats of Antarctic Dry Valleys streams as a natural model ecosystem, we were able to show that nematodes and tardigrades living in these mats have gut microbiomes that are both distinct from each other and from the surrounding microbial community they inhabit. Although at the bacterial phylum level, similar trophic level (bacterial-feeding) but phylogenetically distinct microinvertebrates (*P. murrayi* and tardigrades) had more similar microbiomes than the two phylogenetically closer (*P. murrayi* and *E. antarcticus*) but distinct trophic level (bacterivorous and omnivorous) nematodes, host identity was the most important factor of assembly at more precise levels of taxonomic classification. However, an examination of additional species representing the full range of trophic and phylogenetic diversity is required to fully support this idea.

## Methods

### Sample collection and DNA processing

Cyanobacterial mats were collected from four seasonally active streams (1. Canada, 2. Bowles Creek, 3. Delta, 4. Von Guerard) in the Lake Fryxell Basin (77°36′21″S 163°07′32″E) in Taylor Valley, Antarctica (Fig. 1). Stream length varied from 0.49 km (Bowles Creek) to 5.89 km (Delta Stream) with an average length of 3.2 ± 2.3 km. Canada Stream and Bowles Creek both flow from the Canada Glacier located within the Asgard Range, while Delta Stream and Von Guerard Stream flow from glaciers located within the Kukri Hills. Within each stream, one representative circular plot (2 m radius) with both “black” and “orange” mat types was randomly selected upstream of any flow gauges. In January 2019, three replicates of each mat type were collected by cutting out one 7 cm × 7 cm piece with a sterile scalpel and placing each into sterile 100 mL glass bottles for a total of 24 mat samples (4 streams × 2 mat types × 3 replicates). Samples were frozen at − 20 °C, transported to the University of Florida, and stored at − 20 °C until processing. Mats were slowly defrosted to 4 °C (10 °C increase every 24 h) and examined in sterile Petri dishes under a dissecting microscope. Microinvertebrate individuals visually identified as *Eudorylaimus antarcticus* (n = 88) (currently undergoing taxonomic revaluation), *Plectus murrayi* (n = 176), and tardigrades (n = 94) were collected with a metal pick before being physically moved and agitated through 3 washes of cold sterile water. Previous studies have established similar processes to effectively remove externally adherent microorganisms^22,32^. Following washing, single microinvertebrate individuals were placed in separate 250 μL microcentrifuge tubes containing 25 μL of lysis buffer (800 μg/mL proteinase K, 0.2 M NaCl, 0.2 M Tris–HCL pH 8.0, 1% beta-mercaptoethanol)^58^. Tubes were inspected under a dissecting scope to confirm that only a single microinvertebrate was placed into the lysis buffer. Negative control tubes (n = 7) of lysis buffer, without any microinvertebrates, were prepared in the same manner. Total host and microbiome DNA was extracted by incubating tubes at 65 °C for 120 min to maximize activity of the proteinase K enzyme, followed by 100 °C for 10 min to inactivate it. Substrate mat DNA was extracted from 300 μL of mixed mat slurry using a DNeasy PowerSoil Kit (QIAGEN) according to manufacturer’s protocols. High-throughput metabarcoding was used to characterize bacterial and eukaryotic microbial communities present within microinvertebrate individuals and the mat substrate they were isolated from. Primers and PCR conditions from the Earth Microbiome Project were used to amplify 16S (515F/926R) and 18S (1391f./EukBr) rRNA gene markers^59^ (Supplemental Methods S1). PCR product was then visualized using gel electrophoresis to confirm successful amplification. Three PCR replicates (each amplified using 1μL of DNA template) with negative controls were pooled and sent to the Hubbard Center for Genome Studies, University of New Hampshire for the attachment of indexes (using Golay barcodes)^59^, library preparation, and paired-end sequencing on an Illumina HiSeq 2500 (2 × 250 bp) (Illumina Inc., CA, USA).

### Bioinformatics and community processing

Following read demultiplexing by the sequencing facility, processing of reads was done using QIIME2^60^. First, adapter and primer sequences were removed with cutadapt^61^ and quality filtering was performed in QIIME2 with the quality-filter plugin to trim reads by removing base pairs falling below an average quality score of 30. Reverse 16S reads were discarded due to an insufficient length after quality filtering and unpaired forward reads (196 bp) were denoised to create 100% similarity ASVs using the DADA2 pipeline^62^. Forward and reverse 18S reads were trimmed to 125 bp before joining within DADA2 to create 100% ASVs with an average length of 131 bp and maximum of 172 bp. For both pipelines, chimeras were removed using the built in DADA2 algorithms^62^. Taxonomy was assigned to each ASV using the assign_taxonomy.py script with BLAST in QIIME1.9^63^, comparing against the SILVA v138 database for 16S and SILVA v111 for 18S, with both filtered to remove all reference sequences identified as “uncultured”^64^. Community filtering was conducted using the *phyloseq* package^65^. Non-bacterial sequences in the 16S ASV table and non-eukaryotic sequences in the 18S ASV table were removed. In addition, 18S sequences with hits to the specific microinvertebrate host were removed only from that host’s ASV table, as were 18S ASVs with poor assignments (i.e., below 90% of query coverage and 95% ID) removed from all hosts. Sequences identified in negative controls were subtracted from experimental samples. Due to the uncertain nature of the cyanobacterial clade of *Phormidum/Phormidesmis*^66–68^*,* reads assigned to *Phormidesmis* were assigned to *Phormidium*. Finally, samples with less than 100 total 16S reads and 100 18S reads were discarded based on species rarefaction curves reaching a horizontal asymptote. Furthermore, 9 additional samples of *E. antarcticus* were removed as extreme outliers of uncharacteristically low diversity. After processing and filtering, 4,233,899 bacterial reads and 1,040,761 eukaryotic reads were recovered across a total of 251 microinvertebrate (52 for *E. antarcticus*, 110 for *P. murrayi*, and 89 for tardigrades) and 24 mat samples. For simplicity we define and refer to a “microbiome” as the entire detected microbial community within a sample but recognize that further research may distinguish between resident and transient organisms within the gut.

### Statistics and visualization

Statistics were performed in R Version 3.6.1^69^ (http://www.r-project.org). Alpha diversity metrics (i.e., ASV Richness, Simpson’s, Shannon’s, and Faith’s Phylogenetic Diversity) were calculated with Hill Numbers using *hill_taxa* from the *hillR* package^70^. The effects of community type (mat, *E. antarcticus*, *P. murrayi*, Tardigrada), mat types (black, orange), streams (Canada, Bowles Creek, Delta, Von Guerard), and selected interactions on different measures of alpha diversity were tested using the same general linear model (GLM) with a standard normal distribution (AlphaDiversityMetric ~ Community*Mat*Stream). The significance of the tested variables in the models were evaluated by P and χ^2^ using a type II sum of squares ANOVA. All models were constructed for accuracy and selected based on examining residuals with the DHARMa package^71^. Tukey's HSD was used to compare significance between host identities of different communities (e.g., black mats compared to *P. murrayi* from orange mats, tardigrades from orange mats compared to tardigrades from black mats). Overall community compositional differences among mats as well as among microinvertebrate microbiomes were based on separate Bray Curtis dissimilarity matrices (one for only mats and one for only microinvertebrates) in order to more accurately evaluate the importance (R^2^) of examined factors to each group and were tested using permutational analysis of variance (PERMANOVA) with 9,999 permutations with the *adonis* function in *vegan* 2.5–7^72^, and significance evaluated using P and *F* values. The PERMANOVA for mats was run using (DistanceMatrix ~ Mat*Stream), and for microinvertebrates with (DistanceMatrix ~ HostID*Mat*Stream). Ordinations were visualized using NMDS ordination plots made with the *distance* function within *phyloseq*^65^. Generalized linear mixed models (GLMMs) constructed with *glmmTMB* from the *glmmTMB* package were performed to test the effect of streams (Canada, Bowles Creek, Delta, Von Guerard), mat types (black, orange), community types (mat, *E. antarcticus*, *P. murrayi*, tardigrade), and their interactions on the relative abundances of selected bacterial phyla and genera, with all factors as fixed effects and stream as an additional random effect along with a beta distribution to account for overdispersion^73^ (RelativeAbundance ~ Community*Mat + Mat*Stream + Community*Stream + (1|Stream)). The significance of the tested variables in GLMM models were evaluated by P and χ^2^. To identify the most important taxa to microbial communities at the phylum level, indicator species analysis was conducted with the *indicspecies* package with 9,999 permutations^74^. In addition, linear discriminant analysis effect size (LEfSe) was performed down to the genus level^75^. The LEfSe algorithm identifies characteristic taxa that are both mathematically (Kruskal–Wallis sum-rank test *P* < 0.05) and biologically informative through the use of consistency assessments (unpaired Wilcoxon rank-sum tests *P* < 0.05) and effect size (LDA)^75^. LEfSe provides a taxonomically ranked examination of microbial communities that is not possible with other statistical tools, as for example LEfSe can highlight a family as indicative of a host microbiome, but not the genus within it or the order above it. For our analyses, we applied an LDA effect size score of 4 using the one-against-all method and otherwise default LEfSe parameters on the Huttenhower lab Galaxy server^75^ (https://huttenhower.sph.harvard.edu/). The LEfSe cladogram was exported from the Huttenhower lab Galaxy server and modified in Adobe illustrator for clarity. Initial prediction of the functional profile within microbial communities was performed using PICRUSt2^76^ and tested using the PERMANOVA equations described above, but was not the focus of this study given the limitations of using amplicon data for predicting function^77^ and a limited database of Antarctic bacterial genomes. Sampling map was created using ArcGIS. All other figures were created in R using *ggplot2*.

## Supplementary Information

Supplementary Information.

## Data Availability

Raw reads are available at the NCBI Sequence Read Archive with the project ID PRJNA799934 (https://www.ncbi.nlm.nih.gov/Traces/study/?acc=PRJNA799934). Documented code for the full bioinformatic pipeline, figure creation, and statistical analysis is available at http://www.wormsetal.com/antarcticstreamgutmicrobiomes and www.github.com/WormsEtAl/AntarcticStreamGutMicrobiomes.
