# Supplementary material for: Host identity is the dominant factor in the assembly of nematode and tardigrade gut microbiomes in Antarctic Dry Valley streams
Source: Sci Rep. 2022 Nov 29;12:20118. doi: 10.1038/s41598-022-24206-5 (PMC9709161; doi:10.1038/s41598-022-24206-5)
Supplement: Supplementary file 1 — Supplementary Information. [file 41598_2022_24206_MOESM1_ESM.docx]

**Title:** Host identity is the dominant factor in the assembly of nematode and tardigrade gut microbiomes in Antarctic Dry Valley streams

**Author list:** J. Parr McQueen^1^, Kaitlin Gattoni^1^, Eli M.S. Gendron^1^, Steven K. Schmidt^2^, Pacifica Sommers^2^, and Dorota L. Porazinska^1^

1 Department of Entomology and Nematology, University of Florida, FL 32611

2 Department of Ecology and Evolutionary Biology, University of Colorado Boulder, CO 80303

**Supplemental Information**

Supplemental Table S1. Differences in a. bacterial alpha diversity (Richness, Shannon’s, Simpson’s, Faith’s PD) using a general linear model (GLM) with the equation glm(AlphaDiversityMetric~ Community*Mat*Stream, family=gaussian()). Comparisons included: Community type (Mat, *E. antarcticus, P. murrayi*, Tardigrada), Mat type (black or orange), Stream (Canada, Bowles Creek, Delta, Von Guerard), as well as their interactions. Statistically significant differences are highlighted in bold, as determined by p-values (P) and the likelihood ratio chi-square test (χ^2^). b. Means and standard error of the mean (SE) of alpha diversity metrics (Richness, Shannon’s, Simpson’s, Faith’s PD) for bacterial communities. Alpha diversity metrics were based on ASVs and calculated with Hill Numbers.

| **a.** | | | | |  | Richness | | Shannon's Index | | Simpson’s Index | | Faith's PD | |
| --- | --- | --- | --- | --- | --- | --- | --- | --- | --- | --- | --- | --- | --- |
|  | | |  | | Df | χ^2^ | P | χ^2^ | P | χ^2^ | P | χ^2^ | P |
|  | | | Community | | 3 | **676.87** | **<0.01** | **231.37** | **<0.01** | **58.21** | **<0.01** | **705.07** | **<0.01** |
| Mat | | | | | 1 | 0.06 | 0.80 | 0.03 | 0.87 | 0.03 | 0.85 | 0.21 | 0.65 |
| Stream | | | | | 3 | 3.07 | 0.38 | **11.47** | **0.01** | **14.35** | **<0.01** | 2.91 | 0.41 |
| Community*Mat | | | | | 3 | 2.78 | 0.43 | 3.63 | 0.30 | 5.79 | 0.12 | 3.17 | 0.37 |
|  | | Community*Stream | | | 9 | **27.88** | **<0.01** | **62.24** | **<0.01** | **47.44** | **<0.01** | **23.59** | **0.01** |
|  | | | Mat*Stream | | 3 | 4.85 | 0.18 | 2.50 | 0.48 | 3.01 | 0.39 | 4.72 | 0.19 |
|  | Community*Mat*Stream | | | | 7 | 11.4 | 0.12 | 8.7 | 0.27 | 3.47 | 0.84 | 8.26 | 0.31 |
|  | | |  | |  |  |  |  |  |  |  |  |  |
|  | | |  | |  |  |  |  |  |  |  |  |  |
| **b.** | | | | |  | Richness | | Shannon's Index | | Simpson’s Index | | Faith's PD | |
|  | | | | Mat | N | Mean | SE | Mean | SE | Mean | SE | Mean | SE |
| Mat | | | | Black | 12 | 440.40 | 59.74 | 78.7 | 17.97 | 17.92 | 5.32 | 27.17 | 2.84 |
|  |  |  |  | Orange | 12 | 483.42 | 41.44 | 96.11 | 20.56 | 28.23 | 8.84 | 29.57 | 2.03 |
| *E. antarcticus* | | | | Black | 23 | 12.52 | 3.50 | 7.79 | 2.38 | 5.83 | 1.61 | 2.20 | 0.43 |
|  |  |  |  | Orange | 29 | 5.29 | 1.13 | 3.14 | 0.49 | 2.49 | 0.36 | 1.25 | 0.20 |
| *P. murrayi* | | | | Black | 50 | 28.46 | 3.90 | 9.78 | 1.71 | 6.43 | 1.20 | 3.81 | 0.31 |
|  |  |  |  | Orange | 60 | 28.95 | 5.76 | 10.96 | 1.88 | 6.35 | 0.81 | 3.92 | 0.43 |
| Tardigrada | | | | Black | 51 | 94.73 | 14.12 | 21.50 | 3.64 | 10.48 | 1.60 | 7.71 | 0.77 |
|  |  |  |  | Orange | 38 | 90.11 | 12.98 | 18.49 | 2.98 | 9.29 | 1.24 | 7.10 | 0.69 |

Supplemental Table S2. Differences in a. eukaryotic alpha diversity (Richness, Shannon’s, Simpson’s, Faith’s PD) using a general linear model (GLM) with the equation glm(AlphaMetric~ Community*Mat*Stream, family=gaussian()). Comparisons included: Community type (Mat, *E. antarcticus, P. murrayi*, Tardigrada), Mat type (black or orange), Stream (Canada, Bowles Creek, Delta, Von Guerard), as well as their interactions. Statistically significant differences are highlighted in bold, as determined by p-values (P) and the likelihood ratio chi-square test (χ^2^). b. Means and standard error of the mean (SE) of alpha diversity metrics (Richness, Shannon’s, Simpson’s, Faith’s PD) for eukaryotic communities. Alpha diversity metrics were based on ASVs and calculated with Hill Numbers.

| **a.** | |  | | Richness | Shannon's Index | | Simpson’s Index | | Faith's PD | |
| --- | --- | --- | --- | --- | --- | --- | --- | --- | --- | --- |
|  |  | Df | χ^2^ | P | χ^2^ | P | χ^2^ | P | χ^2^ | P |
| Community | | 3 | **1112.86** | **<0.01** | **45.90** | **<0.01** | **24.59** | **<0.01** | **1877** | **<0.01** |
| Mat | | 1 | 1.45 | 0.23 | **3.57** | **0.06** | 2.10 | 0.15 | 0.45 | 0.50 |
| Stream | | 3 | **9.82** | **0.02** | 4.45 | 0.22 | 5.86 | 0.12 | **10.18** | **0.02** |
| Community*Mat | | 3 | 3.43 | 0.33 | **8.58** | **0.04** | 3.05 | 0.38 | 3.24 | 0.36 |
| Community*Stream | | 9 | **174.23** | **<0.01** | **35.87** | **<0.01** | **17.69** | **0.04** | **136.86** | **<0.01** |
| Mat*Stream | | 3 | **8.61** | **0.03** | 4.42 | 0.22 | 3.05 | 0.38 | **8.48** | **0.04** |
| Community*Mat*Stream | | 7 | **76.56** | **<0.01** | **22.06** | **<0.01** | 6.96 | 0.43 | **20.70** | **<0.01** |
|  |  |  |  |  |  |  |  |  |  |  |
| **b.** | |  | Richness | | Shannon's Index | | Simpson’s Index | | Faith's PD | |
|  | Mat Type | N | Mean | SE | Mean | SE | Mean | SE | Mean | SE |
| Mat | Black | 12 | 77.75 | 16.99 | 11.46 | 3.43 | 5.61 | 1.36 | 13.18 | 1.58 |
|  | Orange | 12 | 69.58 | 5.17 | 5.98 | 0.67 | 2.98 | 0.25 | 13.84 | 0.73 |
| *E. antarcticus* | Black | 23 | 5.38 | 0.62 | 3.49 | 0.26 | 2.86 | 0.20 | 1.51 | 0.12 |
|  | Orange | 29 | 3.96 | 0.50 | 2.69 | 0.28 | 2.36 | 0.25 | 1.35 | 0.20 |
| *P. murrayi* | Black | 50 | 5.13 | 0.72 | 4.26 | 0.57 | 3.77 | 0.48 | 1.40 | 0.15 |
|  | Orange | 60 | 4.19 | 0.51 | 3.51 | 0.35 | 3.21 | 0.31 | 1.08 | 0.11 |
| Tardigrada | Black | 51 | 6.89 | 0.91 | 5.98 | 0.77 | 5.38 | 0.68 | 1.71 | 0.21 |
|  | Orange | 38 | 7.65 | 1.29 | 6.15 | 0.99 | 5.27 | 0.82 | 1.97 | 0.29 |

Supplemental Table S3. Differences in a. bacterial and b. eukaryotic community composition of mat communities using a PERMANOVA with the equation adonis(DistanceMatrix ~ Mat*Stream, permutations = 9999). Comparisons included Mat type (black, orange), Stream (Canada, Bowles Creek, Delta, Von Guerard), and their interactions. Factors explaining the largest variation (R^2^) of each model are underlined.

|  |  | a. Bacterial Mat Communities | | | b. Eukaryotic Mat Communities | | |
| --- | --- | --- | --- | --- | --- | --- | --- |
|  |  |  |  |  |  |  |  |
|  | Df | *F* | P | R^2^ | *F* | P | R^2^ |
| Mat | 1 | 3.94 | < 0.01 | 0.10 | 2.37 | 0.03 | 0.05 |
| Stream | 3 | 4.04 | < 0.01 | 0.32 | 6.18 | < 0.01 | 0.41 |
| Mat:Stream | 3 | 1.69 | < 0.01 | 0.15 | 2.87 | < 0.01 | 0.19 |

Supplemental Table S4. Differences in a. bacterial and b. eukaryotic community composition of microinvertebrate microbiomes using a PERMANOVA with the equation adonis(DistanceMatrix ~ HostID*Mat*Stream, permutations = 9999). Comparisons included Community type (*E. antarcticus, P. murrayi*, Tardigrada), Mat type (black, orange), Stream (Canada, Bowles Creek, Delta, Von Guerard), and their interactions. Factors explaining the largest variation (R^2^) of each model are underlined.

|  |  | 1. Bacterial   Microinvertebrate Microbiomes | | | 1. Eukaryotic Microinvertebrate   Microbiomes | | |
| --- | --- | --- | --- | --- | --- | --- | --- |
|  |  |  |  |  |  |  |  |
|  | Df | *F* | P | R^2^ | *F* | P | R^2^ |
| HostID | 2 | 18.12 | < 0.01 | 0.14 | 3.95 | < 0.01 | 0.04 |
| Mat | 1 | 2.11 | < 0.01 | 0.01 | 1.87 | < 0.01 | < 0.01 |
| Stream | 3 | 4.01 | < 0.01 | 0.04 | 1.78 | < 0.01 | 0.03 |
| HostID*Mat | 2 | 1.38 | 0.04 | 0.01 | 1.18 | 0.11 | 0.01 |
| HostID*Stream | 6 | 0.80 | < 0.01 | 0.04 | 1.07 | 0.2 | 0.02 |
| Mat*Stream | 3 | 0.67 | < 0.01 | 0.02 | 1.20 | 0.05 | 0.01 |
| HostID*Mat*Stream | 4 | 0.48 | < 0.01 | 0.02 | 1.33 | < 0.01 | 0.02 |

Supplemental Table S5: Means and standard error of the mean (SE) for relative abundance of selected taxa (a. Cyanobacteria, Bacteroidota, Proteobacteria, b. *Larkinella*, *Nostoc*, and c. Fungi, Metazoans, Miscellaneous Eukaryotes) within communities. Abundances are reported as proportion of the entire community (i.e., 0-1).

| a. |  |  | Cyanobacteria | | Bacteroidota | | Proteobacteria | |
| --- | --- | --- | --- | --- | --- | --- | --- | --- |
|  | Mat Type | N | Mean | SE | Mean | SE | Mean | SE |
| Mat | Black | 12 | 0.48 | 0.05 | 0.14 | 0.02 | 0.27 | 0.02 |
|  | Orange | 12 | 0.45 | 0.05 | 0.15 | 0.01 | 0.25 | 0.02 |
| *E. antarcticus* | Black | 23 | 0.04 | 0.03 | 0.19 | 0.04 | 0.63 | 0.06 |
|  | Orange | 29 | 0.13 | 0.06 | 0.07 | 0.04 | 0.74 | 0.13 |
| *P. murrayi* | Black | 50 | 0.05 | 0.01 | 0.58 | 0.05 | 0.26 | 0.04 |
|  | Orange | 60 | 0.11 | 0.02 | 0.50 | 0.04 | 0.23 | 0.02 |
| Tardigrada | Black | 51 | 0.06 | 0.02 | 0.54 | 0.04 | 0.28 | 0.02 |
|  | Orange | 38 | 0.04 | 0.02 | 0.58 | 0.04 | 0.27 | 0.03 |
| b. |  |  |  |  |  |  |  |  |
|  |  |  | *Larkinella* | | *Nostoc* | |  |  |
|  | Mat Type | N | Mean | SE | Mean | SE |  |  |
| Mat | Black | 12 | 0.00 | 0.00 | 0.29 | 0.05 |  |  |
|  | Orange | 12 | 0.00 | 0.00 | 0.11 | 0.03 |  |  |
| *E. antarcticus* | Black | 23 | 0.00 | 0.00 | 0.00 | 0.00 |  |  |
|  | Orange | 29 | 0.00 | 0.00 | 0.00 | 0.00 |  |  |
| *P. murrayi* | Black | 50 | 0.40 | 0.05 | 0.00 | 0.00 |  |  |
|  | Orange | 60 | 0.34 | 0.04 | 0.00 | 0.00 |  |  |
| Tardigrada | Black | 51 | 0.00 | 0.00 | 0.00 | 0.00 |  |  |
|  | Orange | 38 | 0.00 | 0.00 | 0.00 | 0.00 |  |  |
| c. |  |  |  |  |  |  |  |  |
|  |  |  | Fungi | | Metazoa | | Miscellaneous Euks | |
|  | Mat Type | N | Mean | SE | Mean | S.E.M. | Mean | SE |
| Mat | Black | 12 | 0.04 | 0.01 | 0.37 | 0.07 | 0.10 | 0.04 |
|  | Orange | 12 | 0.03 | 0.01 | 0.36 | 0.06 | 0.07 | 0.02 |
| *E. antarcticus* | Black | 23 | 0.50 | 0.05 | 0.13 | 0.02 | 0.23 | 0.04 |
|  | Orange | 29 | 0.46 | 0.07 | 0.21 | 0.05 | 0.12 | 0.05 |
| *P. murrayi* | Black | 50 | 0.68 | 0.05 | 0.05 | 0.02 | 0.21 | 0.04 |
|  | Orange | 60 | 0.83 | 0.03 | 0.02 | 0.01 | 0.11 | 0.03 |
| Tardigrada | Black | 51 | 0.80 | 0.04 | 0.03 | 0.01 | 0.13 | 0.03 |
|  | Orange | 38 | 0.77 | 0.05 | 0.02 | 0.01 | 0.17 | 0.05 |

Supplemental Table S6. Indicator species analysis output, run using default parameters at the phylum level among different microbiome types (Mat, *E. antarcticus*, *P. murrayi*, Tardigrada).

| **Indicator species analysis** | |  |  |
| --- | --- | --- | --- |
|  | |  |  |
|  | Significance level (alpha): 0.05 | | |
|  | Total number of Phyla: 34 | | |
|  | Number of Phyla associated to 1 group: 9 | | |
|  | Number of Phyla associated to 2 groups: 1 | | |
|  | Number of Phyla associated to 3 groups: 0 | | |
|  | |  |  |
|  | |  |  |
| Group | | Correlation coefficient  (point biserial) | P Value |
| Group Mat | |  |  |
| Cyanobacteria | | 0.73 | 0.0001 |
| Deinococcota | | 0.45 | 0.0001 |
| Hydrogenedentes | | 0.44 | 0.0001 |
| Myxococcota | | 0.33 | 0.0001 |
| Armatimonadota | | 0.26 | 0.0023 |
| Gemmatimonadota | | 0.22 | 0.0098 |
| Elusimicrobiota | | 0.22 | 0.0108 |
|  | |  |  |
| Group *E. antarcticus* | |  |  |
| Proteobacteria | | 0.63 | 1.00E-04 |
|  | |  |  |
| Group Tardigrada | |  |  |
| Patescibacteria | | 0.26 | 0.0186 |
|  | |  |  |
| Group *P. murrayi*+ Tardigrada | |  |  |
| Bacteroidota | | 0.67 | 1.00E-04 |

Supplemental Table S7. Differences in relative abundance of selected taxa: a. Cyanobacteria, Bacteroidota, Proteobacteria, b. *Larkinella*, *Nostoc*, and c. Fungi, Metazoans, Miscellaneous Eukaryotes using a generalized linear mixed model (GLMM) with the equation glmmTMB(RelativeAbundance ~ Community*Mat+Mat*Stream+ Community*Stream+ (1|Stream), family=beta_family(link="logit"). Comparisons included Cmmunity type (mat, *E. antarcticus, P. murrayi*, Tardigrada), Mat type (black, orange), Stream (Canada, Bowles Creek, Delta, Von Guerard), and their interactions. Statistically significant differences are highlighted in bold, as determined by p-values (P) and the likelihood ratio chi-square test (χ^2^).

|  | | |  | Cyanobacteria | | Bacteroidota | | Proteobacteria | |
| --- | --- | --- | --- | --- | --- | --- | --- | --- | --- |
| a. | | |  |  |  |  |  |  |  |
|  | Factors | Df | | χ^2^ | P | χ^2^ | P | χ^2^ | P |
|  | Community | 3 | | **117.85** | **0.01** | **96.62** | **0.01** | **72.28** | **0.01** |
|  | Mat | 1 | | 3.62 | 0.57 | 1.01 | 0.31 | 0.37 | 0.54 |
|  | Stream | 3 | | **14.98** | **0.01** | **43.86** | **0.01** | 5.93 | 0.11 |
|  | Community*Mat | 3 | | 4.24 | 0.24 | 3.87 | 0.28 | 6.71 | 0.08 |
|  | Mat*Stream | 3 | | **13.96** | **0.01** | **8.33** | **0.04** | 0.89 | 0.83 |
|  | Community*Stream | 9 | | 3.97 | 0.91 | **19.55** | **0.02** | **28.00** | **<0.01** |
| b. | |  | | *Larkinella* | | *Nostoc* | |  |  |
|  | Factors | Df | | χ^2^ | P | χ^2^ | P |  |  |
|  | Community | 3 | | **85.27** | **0.01** | **96.62** | **0.01** |  |  |
|  | Mat | 1 | | 0.08 | 0.78 | 1.01 | 0.31 |  |  |
|  | Stream | 3 | | **15.91** | **<0.01** | **43.86** | **0.01** |  |  |
|  | Community*Mat | 3 | | 0.20 | 0.98 | 3.87 | 0.28 |  |  |
|  | Mat*Stream | 3 | | 3.43 | 0.33 | 8.33 | 0.04 |  |  |
|  | Community*Stream | 9 | | **21.92** | **0.01** | **19.55** | **0.02** |  |  |
|  |  |  | |  |  |  |  |  |  |
| c. |  |  | | Fungi | | Metazoa | | Miscellaneous Euks | |
|  | Factors | Df | | χ^2^ | P | LR Chisq | P | χ^2^ | P |
|  | Community | 3 | | **63.08** | **0.01** | **84.74** | **0.01** | 0.39 | 0.94 |
|  | Mat | 1 | | 3.01 | 0.08 | 0.01 | 0.91 | 3.26 | 0.07 |
|  | Stream | 3 | | **9.00** | **0.03** | 2.51 | 0.47 | 6.96 | 0.07 |
|  | Community*Mat | 3 | | 3.08 | 0.38 | 0.89 | 0.83 | 2.40 | 0.49 |
|  | Mat*Stream | 3 | | 1.92 | 0.59 | 0.06 | 1.00 | 2.08 | 0.56 |
|  | Community*Stream | 9 | | 9.57 | 0.39 | 3.82 | 0.92 | 11.25 | 0.26 |

Supplemental Figure S1. Compositional difference of bacterial communities for cyanobacterial mats as influenced by a. mat type (black or orange) and b. stream (Bowles Creek, Canada Stream, Delta Stream, Von Guerard) based on a Bray Curtis distance matrix, tested using PERMANOVA, and visualized with a NMDS ordination. Eclipses show 95% confidence intervals.


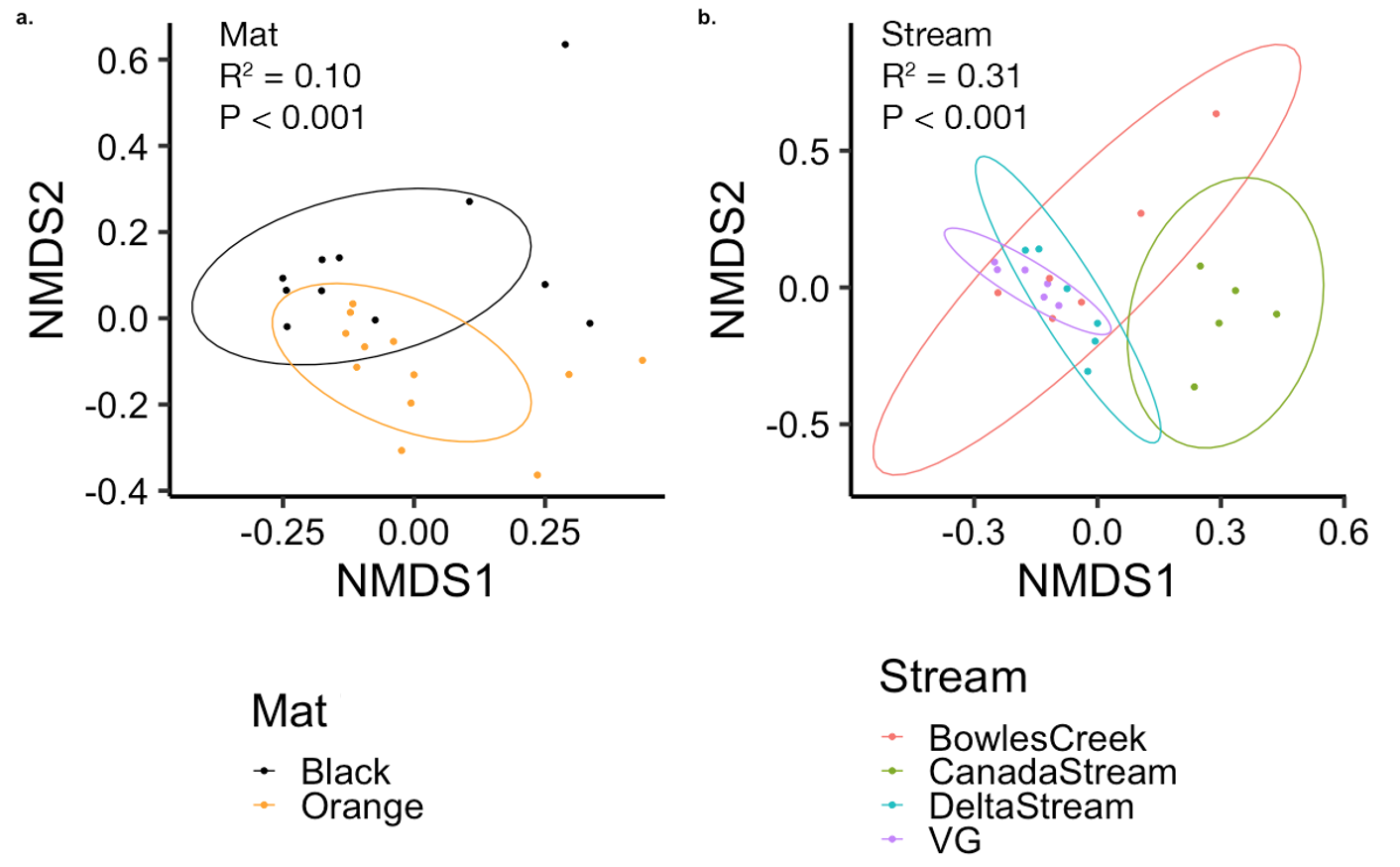


Supplemental Figure S2. Compositional differences of bacterial communities for microinvertebrate gut microbiomes as influenced by a. mat type (black or orange) and b. microinvertebrate type (*E. antarcticus, P. murrayi*, Tardigrada) based on a Bray Curtis distance matrix, tested using PERMANOVA, and visualized with a NMDS ordination. Eclipses show 95% confidence intervals.
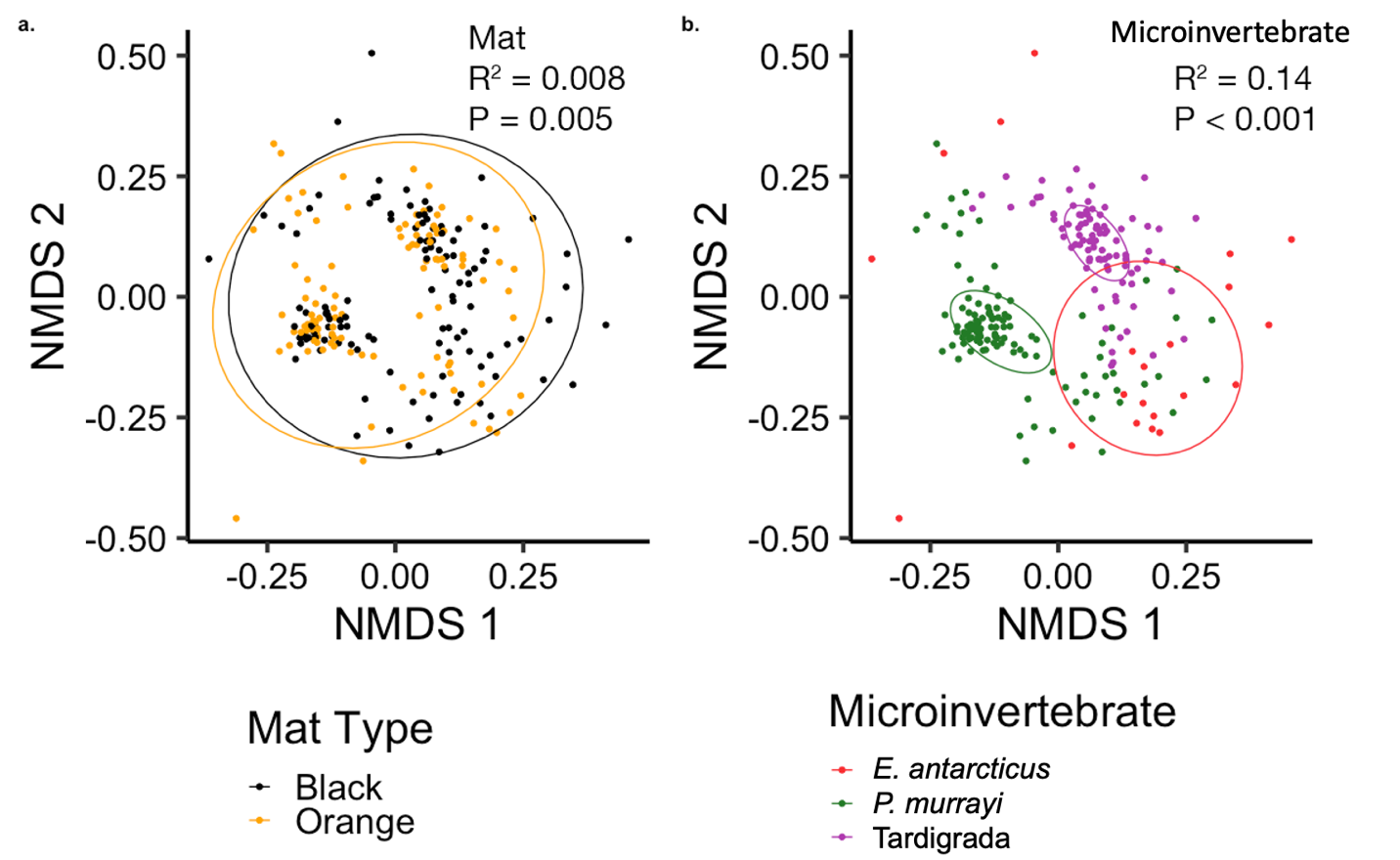
Supplemental Figure S3. Predicted functional profile differences of bacterial communities for cyanobacterial mats as influenced by a. mat type (black or orange) and b. stream (Bowles Creek, Canada Stream, Delta Stream, Von Guerard) using PICRUSt2 analysis and visualized by NMDS. Eclipses show 95% confidence intervals.


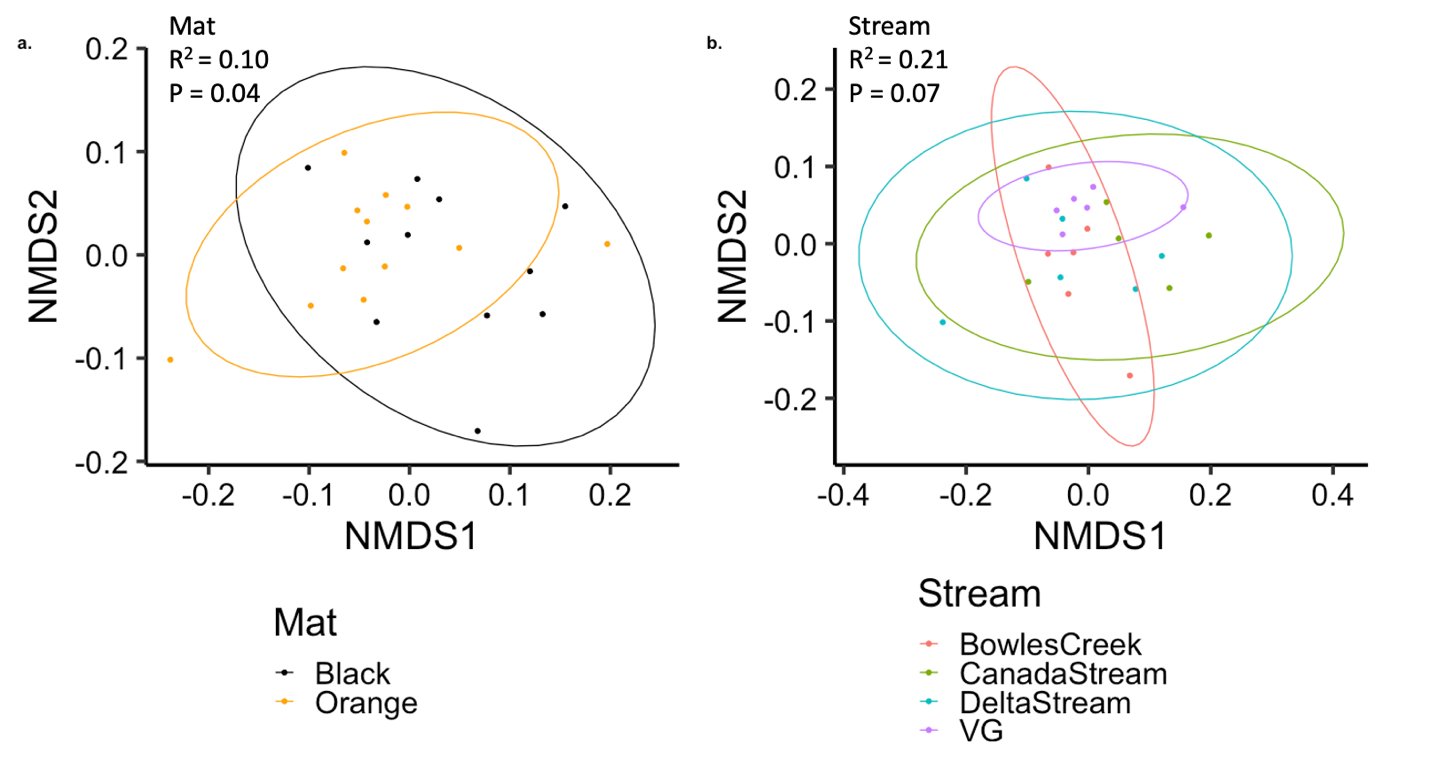


Supplemental Figure S4. Predicted functional profile differences of bacterial communities microinvertebrate gut microbiomes as influenced by a. mat type (black or orange) and b. microinvertebrate type (*E. antarcticus, P. murrayi*, Tardigrada) using PICRUSt2 analysis and visualized by NMDS. Eclipses show 95% confidence intervals.


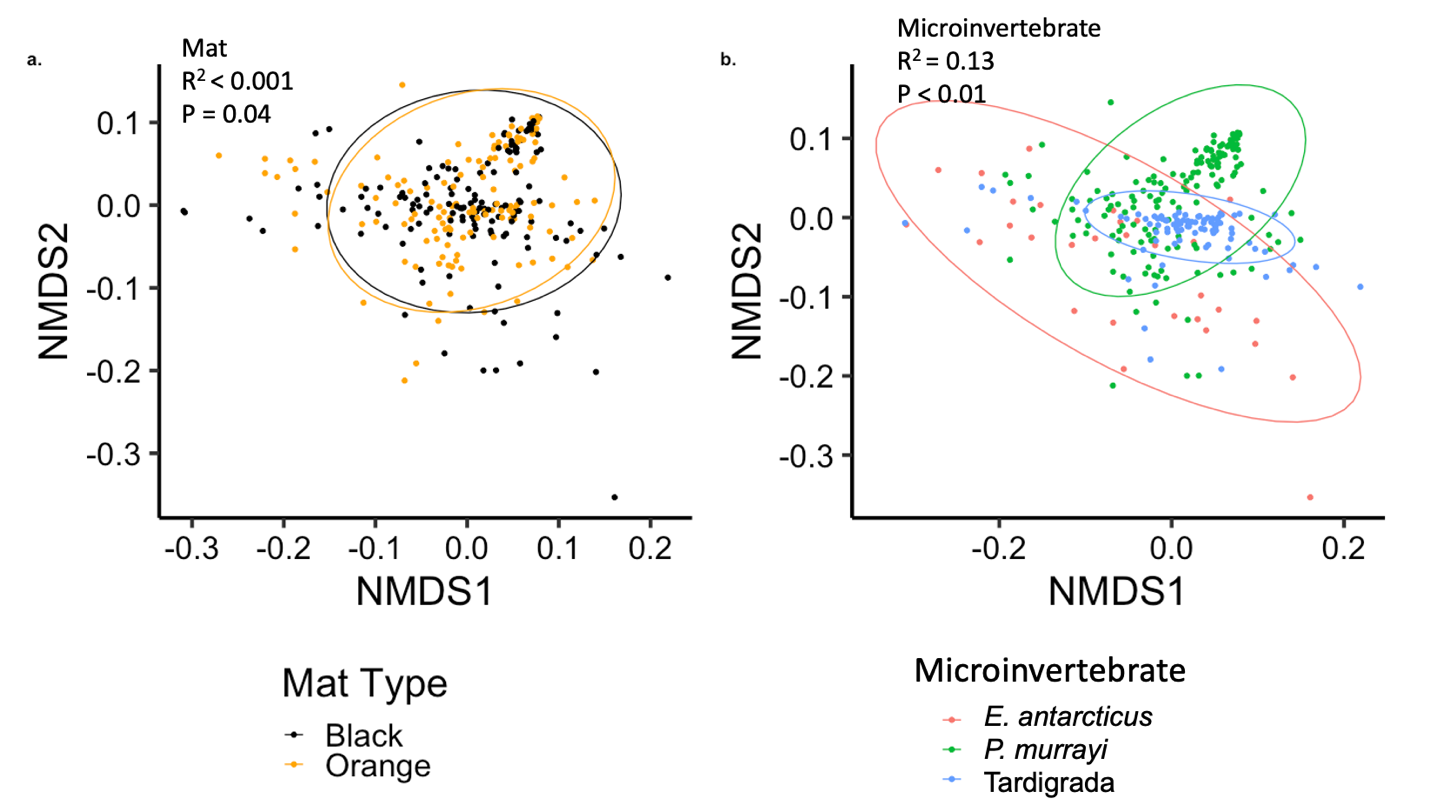


Supplemental Figure S5. Eukaryotic community composition (relative abundance) of mat and microinvertebrate gut microbiomes, for a. the entire eukaryotic community, b. fungal clades, and c. metazoan phyla. Horizontal lines and letters indicate statistical differences (P < 0.05) at the phylum level among the eight communities using GLMM and Tukey post hoc.


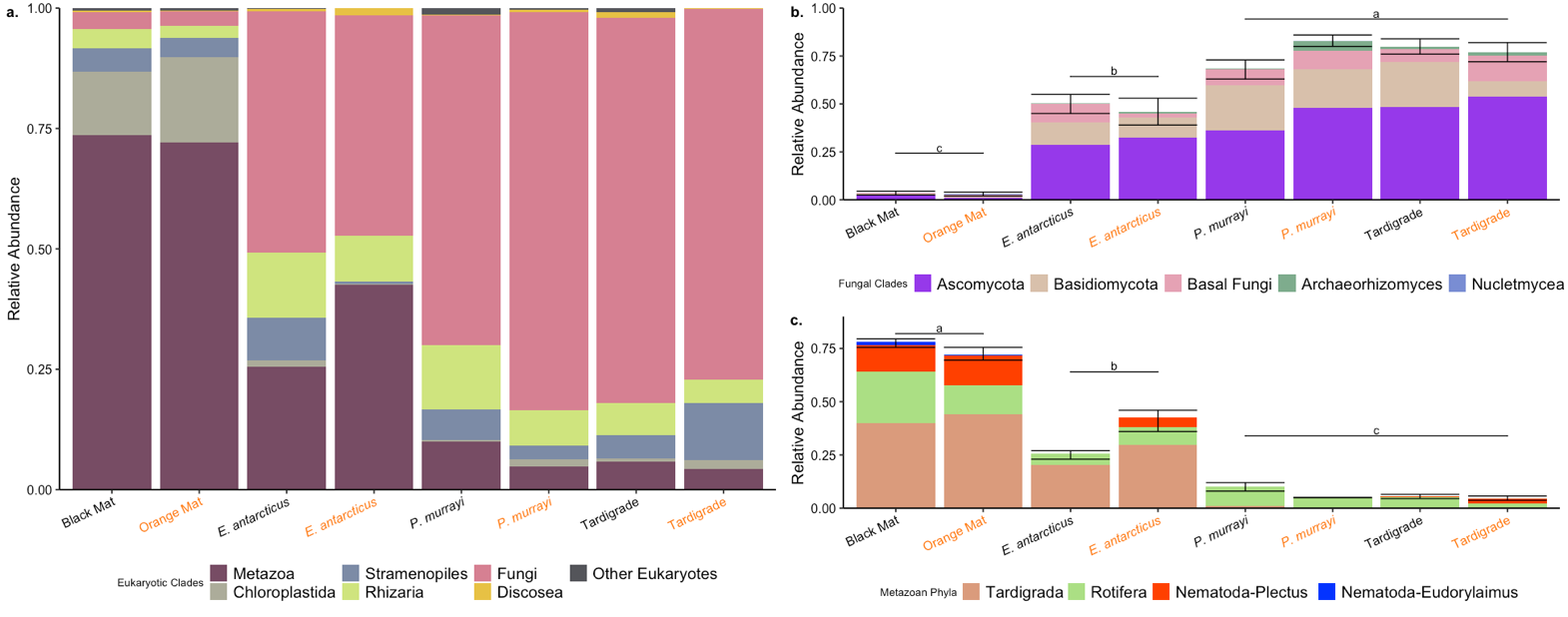


Supplemental Figure S6. Relative abundance of bacterial communities of gut microbiomes from different Tardigrada molecular haplotypes a. bacterial phyla, b. cyanobacterial genera, c. bacteroidota genera, and d. proteobacterial families.

**
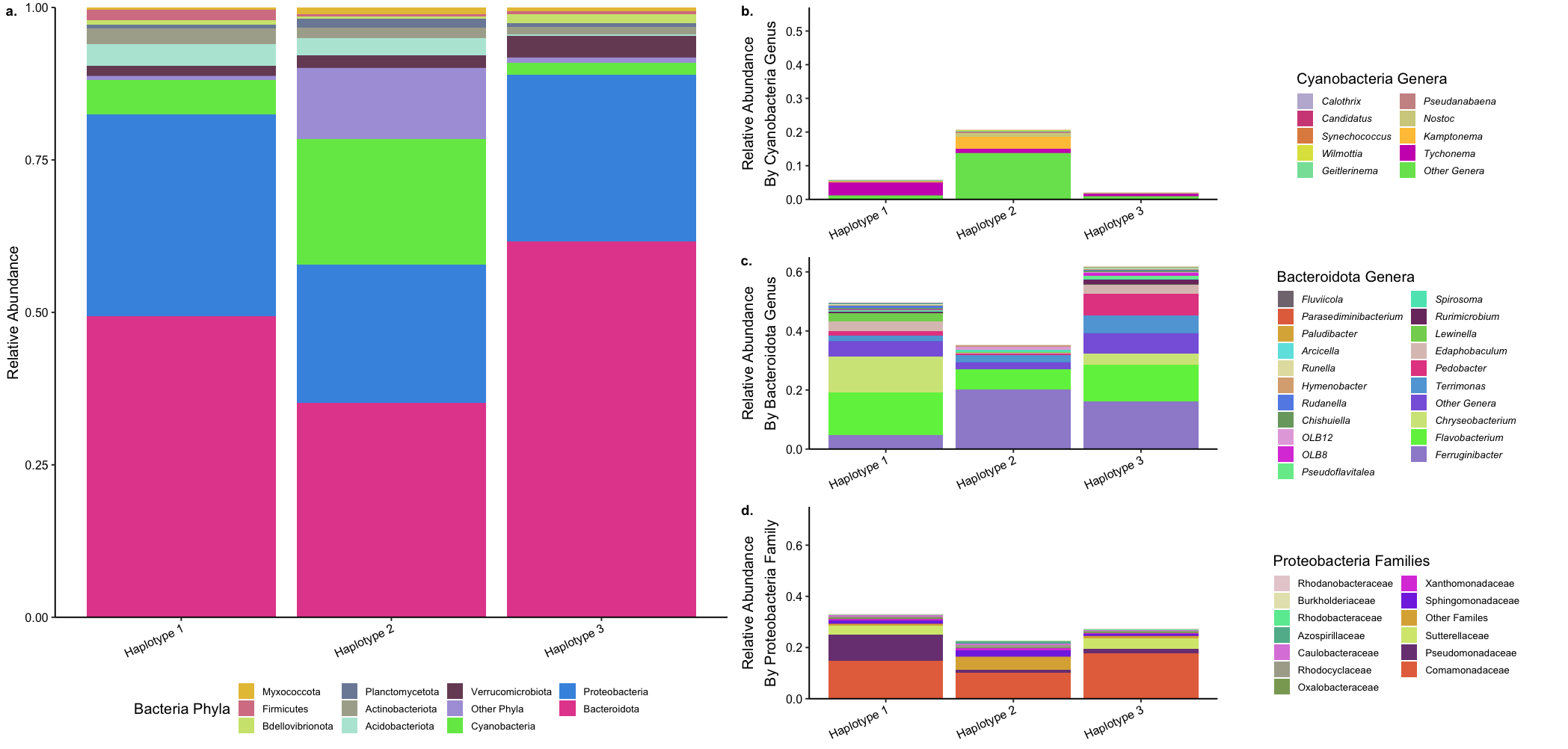
**

Supplemental Figure S7. Compositional difference of bacterial communities for microinvertebrate gut microbiomes of varying observed molecular Tardigrada haplotypes by a. mat type (black or orange) and b. stream (Bowles Creek, Canada Stream, Delta Stream, Von Guerard) based on a Bray Curtis distance matrix, tested using PERMANOVA, and visualized with a NMDS ordination. Eclipses show 95% confidence intervals.


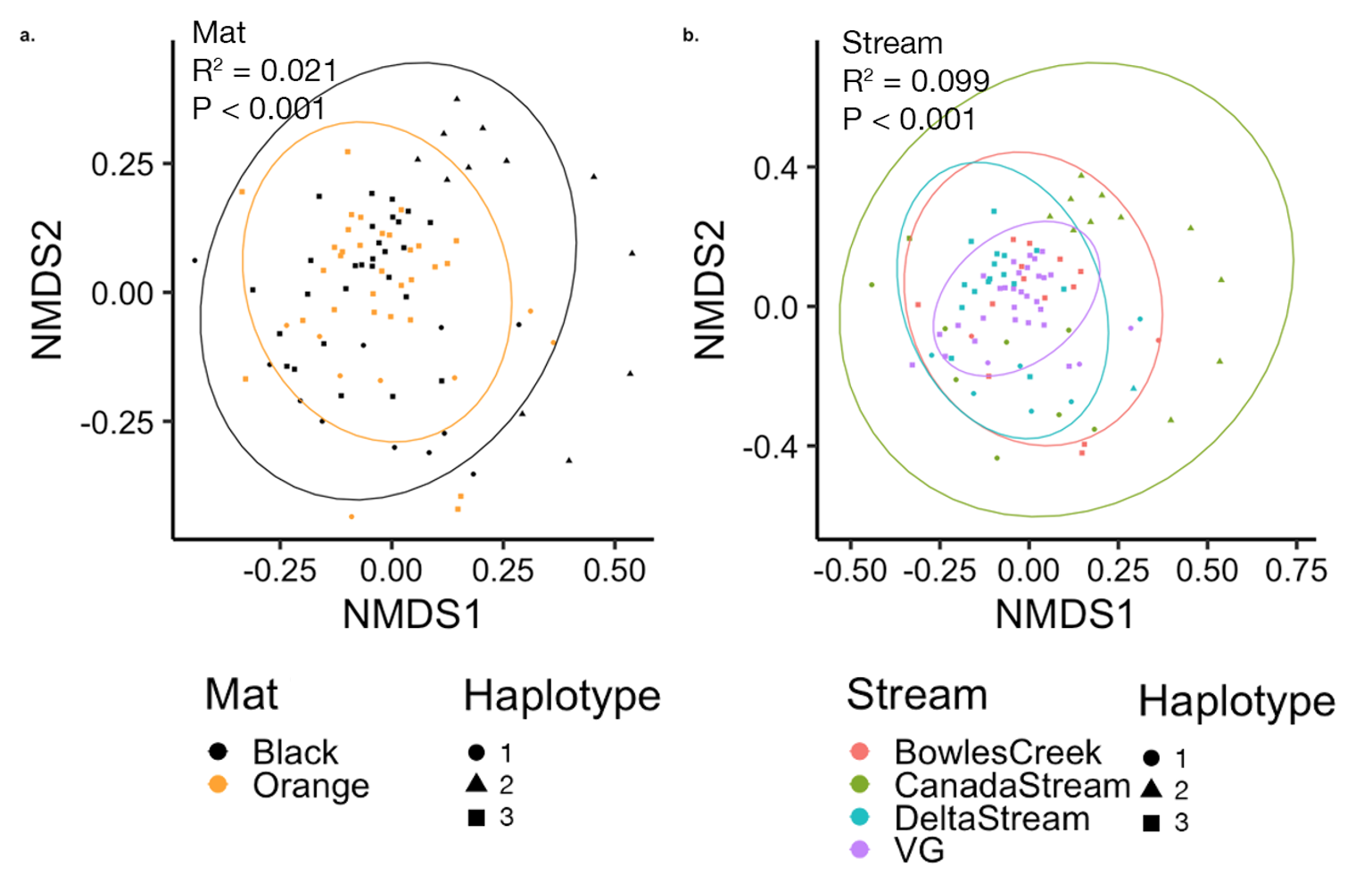


Supplemental Methods S1. PCR conditions used in this study from https://earthmicrobiome.org/.

| 16s PCR Protocol |  |  |  | 18s PCR Protocol |  |  |
| --- | --- | --- | --- | --- | --- | --- |
|  |  |  |  |  |  |  |
| Reagent | Volume |  |  | Reagent | Volume |  |
| PCR-grade water | 13.0 µL |  |  | PCR-grade water | 13.0 µL |  |
| PCR master mix (2x) | 10.0 µL |  |  | PCR master mix (2x) | 10.0 µL |  |
| Forward primer (10 µM) | 0.5 µL |  |  | Forward primer (10 µM) | 0.5 µL |  |
| Reverse primer (10 µM) | 0.5 µL |  |  | Reverse primer (10 µM) | 0.5 µL |  |
| Template DNA | 1.0 µL |  |  | Template DNA | 1.0 µL |  |
| Total reaction volume | 25.0 µL |  |  | Total reaction volume | 25.0 µL |  |
|  |  |  |  |  |  |  |
|  |  |  |  |  |  |  |
|  |  |  |  |  |  |  |
| Temperature | Time | Repeat |  | Temperature | Time | Repeat |
| 94 °C | 3 min |  |  | 94 °C | 3 min |  |
| 94 °C | 45 s | x35 |  | 94 °C | 45 s | x35 |
| 50 °C | 60 s | x35 |  | 57 °C | 60 s | x35 |
| 72 °C | 90 s | x35 |  | 72 °C | 90 s | x35 |
| 72 °C | 10 min |  |  | 72 °C | 10 min |  |
| 4 °C | hold |  |  | 4 °C | hold |  |
|  |  |  |  |  |  |  |
| Notes: Promega Mastermix catalog# M7505 was used for both 18s and 16s | | | | | |  |
